# Transcriptome profiling of *Galaxea fascicularis* and its endosymbiont *Symbiodinium* reveals chronic eutrophication tolerance pathways and metabolic mutualism between partners

**DOI:** 10.1038/srep42100

**Published:** 2017-02-09

**Authors:** Zhenyue Lin, Mingliang Chen, Xu Dong, Xinqing Zheng, Haining Huang, Xun Xu, Jianming Chen

**Affiliations:** 1 School of Life Sciences, Xiamen University; Fujian Collaborative Innovation Center for Exploitation and Utilization of Marine Biological Resources, Xiamen, Fujian 361005, China; 2 State Key Laboratory Breeding Base of Marine Genetic Resources; Key Laboratory of Marine Genetic Resources, Third Institute of Oceanography, State Oceanic Administration; Key Laboratory of Marine Genetic Resources of Fujian Province, Xiamen 361005, China; 3 Laboratory of Marine Chemistry and Environmental Monitoring, Third Institute of Oceanography, State Oceanic Administration, Xiamen, Fujian 361005, China; 4 Laboratory of Marine Biology and Ecology, Third Institute of Oceanography, State Oceanic Administration, Xiamen, Fujian 361005, China

## Abstract

In the South China Sea, coastal eutrophication in the Beibu Gulf has seriously threatened reef habitats by subjecting corals to chronic physiological stress. To determine how coral holobionts may tolerate such conditions, we examined the transcriptomes of healthy colonies of the galaxy coral *Galaxea fascicularis* and its endosymbiont *Symbiodinium* from two reef sites experiencing pristine or eutrophied nutrient regimes. We identified 236 and 205 genes that were differentially expressed in eutrophied hosts and symbionts, respectively. Both gene sets included pathways related to stress responses and metabolic interactions. An analysis of genes originating from each partner revealed striking metabolic integration with respect to vitamins, cofactors, amino acids, fatty acids, and secondary metabolite biosynthesis. The expression levels of these genes supported the existence of a continuum of mutualism in this coral-algal symbiosis. Additionally, large sets of transcription factors, cell signal transduction molecules, biomineralization components, and galaxin-related proteins were expanded in *G. fascicularis* relative to other coral species.

Due to the high risk of land-based sources of pollution, the most biodiverse coral reefs in Southeast Asia have so far been neglected^1^. Beibu Gulf is a semi-enclosed gulf located in the northwest of the South China Sea, which is surrounded by Vietnam, Guangxi, Leizhou Peninsula and Hainan Island. The deteriorating eutrophication in Beibu Gulf due to the increasing nutrient and organic matter fluxes over the past 30 years is one of the major threats to coral reefs^2^. Eutrophication and turbidity enrichment directly affect coral physiology, including photosynthesis, respiration, calcification and reproduction, resulting in coral diseases and the microbial community shift of corals^3^. Most coral species appear to be sensitive to eutrophication^4^, while some corals, such as *Galaxea retiformis* and *Turbinaria mesenterina*, are considered to be more resistant due to a higher trophic plasticity^5^. To comprehensively understand the mechanism of eutrophication tolerance, fundamental studies are needed to shed light on the perception of the eutrophic disturbance and the corals’ holobiont metabolic and molecular resilient regimens.

*Galaxea fascicularis* is a type of massy reef-building coral that is widely distributed in the tropic Indo-Pacific area^6^. *G. fascicularis* is classified into soft and hard types based on nematocyst morphology^7^. Because of genetic differentiation^8^, their morphological characteristics are correlated with the length of the noncoding region between the mitochondrial genes cytb and nad2^9^. A more recent study reported several cryptic types found in *G. fascicularis* based on genetic information from microsatellite loci^8^,^10^. *G. fascicularis* shows intraspecific genetic diversity and connectivity and appears to have the potential for the recovery of populations after disturbance^8^,^10^. Additionally, *G. fascicularis* is relatively resilient to bleaching and sedimentation^11^ and is therefore widely used for comparative analyses of coral responses to thermal, acidulated and light stresses^12^,^13^. Despite the unique and interesting biological features of *G. fascicularis*, molecular information about its responses to stressors is still limited.

Coral bleaching often results from increasing environmental perturbation, which causes corals to lose their vital photosynthetic dinoflagellate symbionts (*Symbiodinium*)^14^. Despite the importance of the mutualism between coral and *Symbiodinium*, it is surprising that very limited information is available about the molecular events underlying the establishment, interaction and collapse of the mutualism under stress. Here, we used a transcriptomic approach to elucidate the transcriptional profiles of native *G. fascicularis* and its endosymbionts in Beibu Gulf of China, and furthermore, we identified specific genes and possible metabolic pathways involved in tolerance to mid to long-term eutrophic stress. The availability of transcriptome sequences and gene annotation enabled us to study the molecular interactions between the host coral and its endosymbiont *Symbiodinium*. Our study demonstrates that the usefulness of the *G. fascicularis* transcriptome will accelerate the understanding of the mechanisms of reef coral resistance on the impacts of anthropogenic global climate changes.

## Results

### Transcriptome assembly and annotation

To globally characterize the transcriptome of *G. fascicularis* and its *Symbiodinium* endosymbionts with enhanced sequence coverage, 12 cDNA libraries were constructed using *G. fascicularis* polys obtained from eastern and western pools of Hainan Island, China. In total, 289,711,812 Illumina paired-end raw reads were generated. After discarding adaptor and low-quality sequences, we obtained 281,021,672 clean reads (accounting for 42.15 Gb). The percentages of clean reads among the raw tags (Q20) ranged from 96.74 to 97.02% (Table S1). The *de novo* assembly of clean reads resulted in 347,084 unigenes in the range of 201–50,485 bp, with an N50 length of 1,259 bp (Fig. S1). In total, 150,771 (43.44%) unigenes were successfully annotated in at least one of the Nr, Nt, Swiss-Prot, KEGG, GO, COG and Pfam public databases (Table S2).

### Dissecting *G. fascicularis* and its *Symbiodinium* endosymbiont transcriptomes

BLASTN analysis of the *G. fascicularis* symbiosis transcriptome against the custom genome database sets of *Nematostella vectensis, Acropora digitifera* and *Symbiodinium* (e-value < 1E-10) indicated that 13,293 (3.83%), 11,098 (3.20%) and 25,030 (7.21%) unigenes had significant matches to the genomes of these species, respectively. Among the remaining 297,664 (85.76%) unigenes, approximately 88,415 (25.47%) of them were annotated in the NCBI nr database. Figure S2 depicts the species distribution of the top BLAST results. Of these 88,415 protein-encoding unigenes, 31,844 (36.02%) matched metazoan genes (*i.e.*, invertebrate and vertebrate) and thus were presumably of coral (*G. fascicularis*) origin^15^. Likewise, 24,189 (27.36%) unigenes were significantly aligned to genes from alveolates, other algae, or green plants. These unigenes were assumed to be of *Symbiodinium* origin (Fig. S3A)^16^. Additionally, approximately 32,382 (36.63%) of these nr database-matching unigenes aligned to bacteria or undetermined taxonomic assignment and were not used in the subsequent analyses.

A previous study has suggested that variations in the GC content are correlated with the origin and evolution of species’ life-history features^17^. The mean GC content corresponding to typical exons for anthozoan cnidarians (the mean GC contents of *A. digitifera* and *Nematostella* are similar) was approximately 40%. In comparison, the mean GC content of the *Symbiodinium* genome is over 50%^18^,^19^,^20^. We further investigated the GC content distribution of all the sequences in the assembly. Two clear peaks at approximately 43 and 55% were detected (Fig. S3B), suggesting that the two peaks possibly resulted from mixed transcriptomes of coral (*G. fascicularis*) and *Symbiodinium*. The peak GC content and N50 in the components of the *G. fascicularis* and *Symbiodinium* origin assembly were approximately 42.96% (N50 length 2,735 bp) and 54.91% (N50 length 1,893 bp), respectively (Table S3). These results were largely congruent with the fact that the two peaks were detected in the whole transcriptome, indicating that we effectively separated *G. fascicularis* and *Symbiodinium*-originated unigenes.

### Transcriptome-wide differential expression in response to eutrophication

#### Eutrophic environment in the west coast of Hainan Island

**Figure 1:**
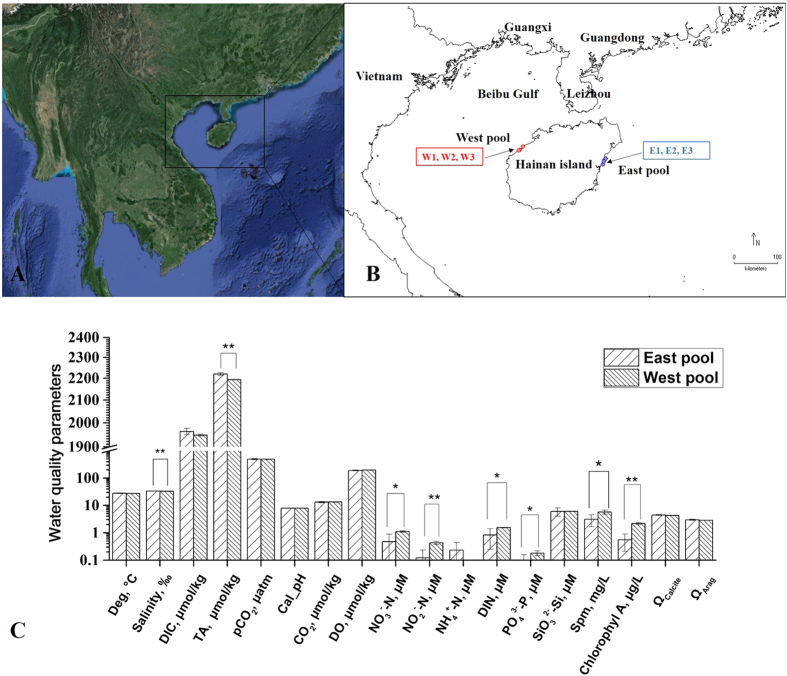
(**A**) Overview of the location of the study areas at Beibu Gulf of the South China Sea. The map was created using ArcGIS 10.3.1 (http://www.esri.com/software/arcgis). (**B**) Location of the coral sampling sites of the western and eastern pool of Hainan island. The map was drawn using DIVA-GIS (Version 7.5.0, http://www.diva-gis.org/) software based on coordinates recorded for each locality with a GPS device. ●: point of *G. fascicularis* samples. (**C**) Mean environmental conditions in eastern and western points of water sampling (sample sizes are n = 13 at the eastern pool and n = 15 at the western pool). Values represent the mean ± SD data for analysing the statistical significance by Student’s t-test. Asterisks indicate significant differences (*p < 0.05, **p < 0.01). Abbreviations are as follows: Deg: Degrees Celsius; DIC: Dissolved inorganic carbon; TA: Total alkalinity; DO: Dissolved oxygen; DIN: Dissolved inorganic nitrogen; SPM: Suspended particulate matter; Ω calcite: calcite saturation state; Ω arag: Aragonite saturation state.

As a prerequisite for this study, we investigated the eutrophication status of the two reef sampling sites (western and eastern pools of Hainan Island) (Fig. 1A,B). There were significant differences in the concentrations of the total alkalinity (TA), dissolved inorganic nitrogen (DIN), NO_3_^−^, NO_2_^−^, PO_4_^−^, suspended particulate matter (SPM) and chlorophyll a between eastern and western pools during the period of our sampling (*p* < 0.05) (Fig. 1C). As expected, high concentrations of dissolved inorganic nitrogen (DIN, NO_3_^−^ and NO_2_^−^), phosphate (PO_4_^−^) and chlorophyll a at western sampling sites reflected eutrophic conditions in western coastal waters^21^. Consistently, many studies have shown that in recent decades, strong human impacts on Beibu gulf areas have resulted in a high abundance of nitrogen and phosphorus signals, SPM and phytoplankton, which may contribute to coastal eutrophication^2^. This process motivated us to explore the molecular basis of coral tolerance to chronic seawater eutrophication.

#### Differential gene expression patterns of coral

**Figure 2:**
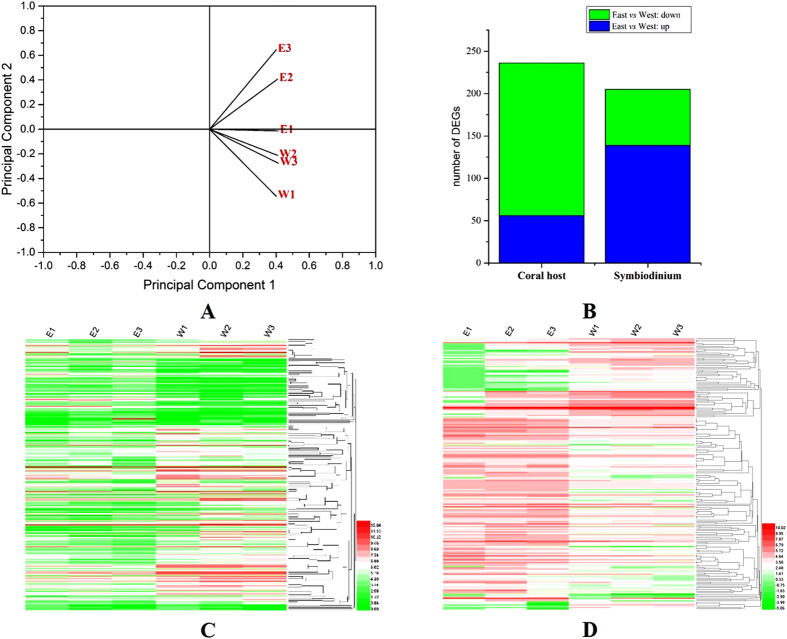
(**A**) Principal component analysis based on the expression level data of the *G. fascicularis* symbioses transcriptome from six sites. Gene expression was computed as FPKM with a pseudocount of 0.01. Principal component analysis was performed in Origin Pro 9.1. The first two principal components explained 95.45% of the total variance. (**B**) Overview of the number of differentially expressed genes. The differentially expressed genes of the *G. fascicularis* host and its endosymbiont *Symbiodinium* were identified by comparing the east and west transcriptome with |log2 fold change| > 1.0 and FDR adjusted p-values ≤ 0.05. Heatmap analysis of differentially expressed genes identified in the *G. fascicularis* host (**C**) and its endosymbiont *Symbiodinium* (**D**). The heatmap was created using heatmap2 in the R statistical computing environment, and the gene expression was characterized as the log2 of the ratio between expression levels in the 6 sites. Higher expression levels are shown in pink, while lower expression levels are shown in green. (E1, E2 and E3: sampling sites in the east pool. W1, W2 and W3: sampling sites in the west pool).

Our transcriptome dataset included the expression levels of unigenes from *G. fascicularis* and its endosymbiont *Symbiodinium*. We performed a principal component analysis (PCA) on the normalized counts for *G. fascicularis* holobiont unigenes to visualize the extent of variation between the two pools and found that the samples were distinctly separated by geographical location between the eastern and western pools (Fig. 2A). Substantial differences in the gene expression patterns of *G. fascicularis* holobionts perhaps represented signs of genetically based or mid- to long-term adaptive acquisition of tolerance to eutrophication in western sites. Coral gene expression data revealed that the expression of 236 unigenes (1.06%) differed significantly between the two sample sets with pristine versus eutrophied conditions (origin: east *vs* west; |log2 fold change| > 1.0 expression difference, FDR–corrected *P* value < 0.05). Of these unigenes, 56 had higher expression levels at the pristine (east) pool, while the remaining 180 unigenes had higher expression levels at the eutrophied (west) pool (Fig. 2B,C). Out of 236 unigenes, 117 were annotated with the Swiss-Prot database (Table S4). Among the annotated genes, we found that many of them encode transcription factors (11 genes), cell signalling proteins (20 genes), innate immunity related factors (e.g., NF-κB and TNF receptor–associated TRAF–type proteins, 4 genes), solute carriers (6 genes), cytochrome P450 (2 genes), components of the TCA cycle (3 genes), lipid or fatty acid synthases (5 genes), and regulators of cell cycle and apoptosis (3 genes). In particular, a large number of genes (43 genes, 24%) with increased expression in the eutrophied pool were involved in innate immunity, cell apoptosis and energy metabolism.

#### Expression variation of different Symbiont types

The *Symbiodinium* types in each coral sample were determined by a molecular approach^22^. The alignment results of the *Symbiodinium* internal transcribed spacer 2 (ITS2) sequences reflected that symbiont types C1, D1a, and D1 were detected, and type C1 was dominant in all colonies examined (Table S5). It is likely that the eutrophied environment did not change the dominant type of *Symbiodinium*.

Our transcriptome data showed that 139 and 66 unigenes were up-regulated and down-regulated in the pristine pool compared to the eutrophied pool, respectively (|log2 FC| > 1.0, FDR–corrected *P* value < 0.05) (Fig. 2B and D). Only 39 of these unigenes were annotated with KEGG terms. The affected genes could be assigned to the biosynthesis of secondary metabolites (6 genes), transportation (3 genes), protein processing in endoplasmic reticulum (2 genes), ribosome (2 genes), photosynthesis (*LHCA1* gene), circadian clock regulation (*CSNK1E* gene) and were associated with physiological plasticity under environmental stressors (4 genes) (Table S6), suggesting that the metabolic and cellular processes of symbionts were also affected under long-term eutrophic stress.

### Functional analysis of the unigene sets originating from *G. fascicularis* and *Symbiodinium*

To investigate the comprehensive transcriptome of the holobiont consisting of *G. fascicularis* and its symbionts from the perspective of systems biology, we first categorized the unigene sets originated from *G. fascicularis* and *Symbiodinium* separately by gene ontology (GO) analysis. We assigned 8,460 and 17,177 unigenes to one or more GO terms for *G. fascicularis* and *Symbiodinium*, respectively (Fig. S4). In comparison, we observed that for most functional categories that are essential for normal cell activities, *Symbiodinium* have lower unigene percentages than the coral host, except for the three categories related to the extracellular matrix, catalytic activity and transporter activity.

**Figure 3:**
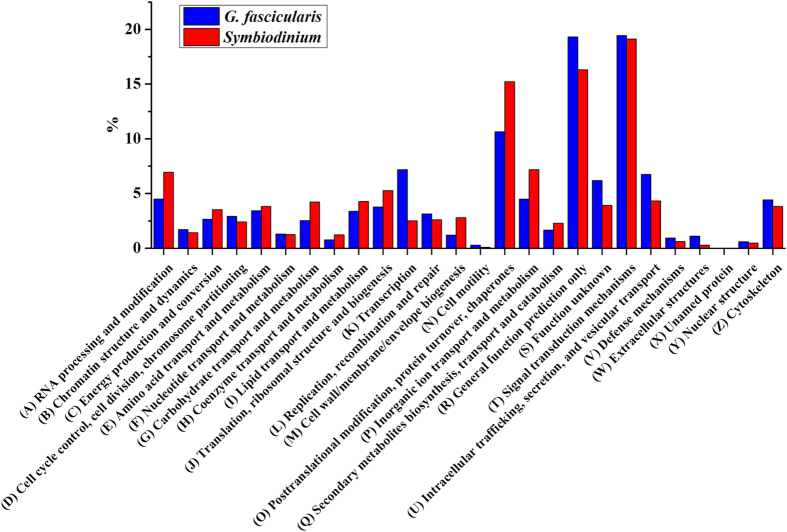
Large-scale comparison of KOG categories assigned to *G. fascicularis* and its *Symbiodinium* symbiont transcriptomes. The enrichment analysis (y-axis) is expressed as the percentage of sequences in the *G. fascicularis* and its *Symbiodinium* symbiont transcriptome sets, respectively.

The unigenes originated from each component were subjected to a search against the KOG database for functional classification. We subdivided 6,752 and 9,307 non-redundant unigenes of *G. fascicularis* and *Symbiodinium*, respectively, into 26 classifications (Fig. 3). Interestingly, *G. fascicularis*-host and *Symbiodinium* had different percentages of unigenes involved in metabolic pathways, suggesting that the metabolic components of *G. fascicularis*-host and *Symbiodinium* had distinct enrichment patterns. Of these pathways, 7 *Symbiodinium* showed much higher percentages than that in *G. fascicularis*-host, including (A) RNA processing and modification, (C) energy production and conversion, (G) carbohydrate transport and metabolism, (I) lipid transport and metabolism, (M) cell wall/membrane/envelope biogenesis, (O) posttranslational modification, protein turnover, chaperones and (P) inorganic ion transport and metabolism. In contrast, several other pathways were particularly abundant in *G. fascicularis*-host, whereas they were represented in much lower percentages in *Symbiodinium*. These pathways included (B) chromatin structure and dynamics, (D) cell cycle control, cell division, chromosome partitioning, (K) transcription, (R) general function prediction and (U) intracellular trafficking, secretion, and vesicular transport.

In addition, KEGG enrichment analysis for *G. fascicularis* and its *Symbiodinium* symbiont transcriptomes was performed. *Symbiodinium* retained very abundant genes involved in nutrient provision to their hosts, which was associated with carbohydrate metabolism, energy metabolism, glycan biosynthesis, lipid metabolism and nucleotide metabolism (Fig. S5). These findings are consistent with a previous report showing that the coral host acquired and utilized sugars and carbon sources for energy metabolism from its *Symbiodinium*^23^. In addition to the above-mentioned metabolic pathways, we were also surprised by the observation that the *Symbiodinium* transcriptome had an ample amount of genes associated with other metabolic pathways, including amino acid metabolism, vitamin and cofactor metabolism, biosynthesis of secondary metabolites and metabolism of terpenoids and polyketides. Many of these genes were not found in the *G. fascicularis*-host transcriptome, which pointed to the genetic complementarity between *G. fascicularis* and *Symbiodinium*.

### Integration of metabolic pathways between coral and *Symbiodinium*

**Figure 4:**
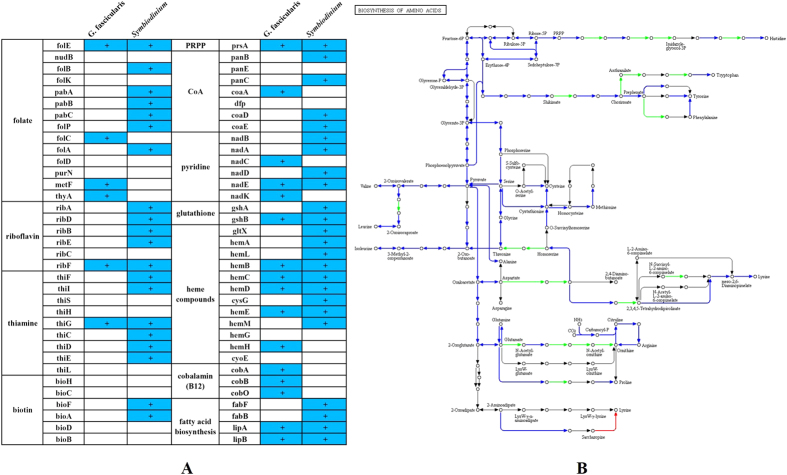
(**A**) Vitamin-related gene expression and retention patterns for the *G. fascicularis* and *Symbiodinium* symbiont transcriptomes. (**B**) The predicted amino acid biosynthesis pathways of *G. fascicularis* and *Symbiodinium*. Blue lines indicate genes present in both the *Symbiodinium* and *G. fascicularis* transcriptome. Red lines indicate genes only present in the *G. fascicularis* transcriptome. Green lines indicate genes only present in the *Symbiodinium* transcriptome. Grey lines indicate genes absent in the *Symbiodinium* and coral (*G. fascicularis*) transcriptome.

The analysis of a metabolic pathway map based on *G. fascicularis* and its *Symbiodinium* symbiont transcriptomes revealed that high integration and interdependence at the metabolic level occurred in the coral-symbiotic system (Fig. S6). Of note, *G. fascicularis* holobiont showed a striking metabolic complementarity in vitamin and cofactor biosynthesis. A large number of the *Symbiodinium* unigene-encoded proteins were predicted to play important roles in the synthesis pathways of a diverse set of vitamins, cofactors, prosthetic groups and other related compounds. However, the paucity of vitamin and cofactor synthesis pathways in the *G. fascicularis* host suggested the possibility that unigenes in *G. fascicularis* and *Symbiodinium* may play distinct and collaborative roles in this process^24^ (Fig. 4A). Furthermore, *G. fascicularis* showed an incomplete fatty acid biosynthetic pathway due to the lack of the β-ketoacyl-ACP synthase I and II genes (*fabF* and *fabB*). These two genes can be found in the *Symbiodinium* based on our transcriptome data. Our data also provided molecular evidence of a mutualistic continuum between coral hosts and *Symbiodinium* in amino acid synthesis. The amino acid synthesis pathways showed a *Symbiodinium*-dominated tendency as the majority of genes participating in these pathways were only found in *Symbiodinium* (Fig. 4B). The coral holobiont had complete pathways for the biosynthesis of most standard amino acids but lacked several functional genes that are important for the biosynthesis of histidine (*hisB* gene is missing), tryptophan (*trp1* gene is missing), tyrosine (*tyrA, tyr B* and *tyrC* genes are missing), and phenylalanine (*tyrA, tyrB* and *tyrC* genes are missing). To test these hypotheses, we investigated whether any critical genes necessary for the biosynthesis of vitamins and cofactors and amino acids were also lost in the *A. digitifera* genome. Indeed, the biosynthesis pathways of vitamins and cofactors and amino acids were also incomplete in *A. digitifera* according to the KEGG annotation, which is consistent with our observation. Additionally, *G. fascicularis* holobiont also showed remarkable integration in the biosynthesis of secondary metabolites (Fig. S7) to produce the diverse and complex compounds required by members of the consortium. However, it is still unclear how the coral host and *Symbiodinium* coordinate their respective systems in the biosynthesis of vitamins and cofactors, fatty acids, amino acids and secondary metabolites to establish mutualistic nutritional benefits.

### Comparative analysis of prominent gene families revealed significant differences among related species

**Figure 5:**
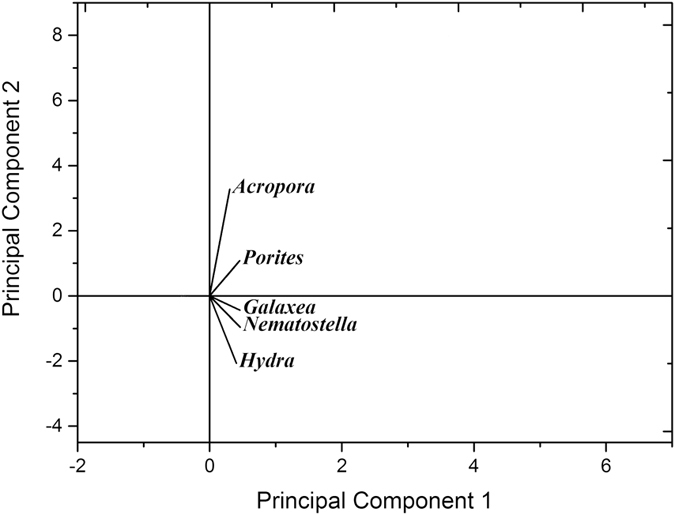
Principal component analysis depicting the correlations between predictor variables and the relative abundance of domain similarities of *G. fascicularis* and other related corals. Principal component analysis performed in Origin Pro 9.1. The first two principal components explain 95.58% of the total variance. Abbreviations are as follows: Acropora: *Acropora digitifera,* Porites: *Porites australiensis,* Galaxea: *Galaxea fasciculari,* Nematostella: *Nematostella vectensis,* Hydra: *Hydra magnipapillata*.

A transcriptome-scale comparison of major gene families in related species suggested that some families were expanded in *G. fascicularis*. PCA allowed the estimation of the depth of the divergence among corals and other *Cnidaria* species. The domain similarities composition of the *G. fascicularis* was closely related to that of *Porites australiensis* and *N. vectensis* among the species examined (Fig. 5). The numbers of predicted transcription factor genes and cell signalling molecules in *G. fascicularis* were significantly higher in comparison with those in *P. australiensis* and *A. digitifera* but lower than that in *N. vectensis* (Tables S7 and S8). We applied a comparative analysis of the predicted biomineralization-related proteins involved in the formation of inorganic skeletons and organic matrices in *G. fascicularis* and other species. The results showed that the number of biomineralization-related proteins in the *G. fascicularis* transcriptome was expanded to 68, compared to 48 in *P. australiensis* and 43 in *A. digitifera* (Table S9). Additionally, there were only 289 unigenes with conserved innate immunity and the apoptosis domain found in the *G. fascicularis* transcriptome (Table S10). The number was significantly less than that in *P. australiensis* (379 unigenes) and *A. digitifera* (833 unigenes).

We further searched for the unigenes and pathways potentially involved in the *G. fascicularis* symbiosis. The symbiosis-associated genes fell into several broad functional categories as listed in Table S11 25. These genes were identified by the presence of characteristic domains in the predicted proteins by BLASTx analysis. These genes were then compared to homologous sequences in publicly available resources (genomes and transcriptomes) for *A. digitifera, A. hyacinthus, A. millepora, A. palmata, M. faveolata, P. astreoides, P. damicornis*, and the more distantly related species *N. vectensis, A. pallida* and *A. viridis*. The symbiosis-associated genes of *G. fascicularis* were more similar to those of the other corals (*A. hyacinthus, A. millepora, P. astreoides* and *A. digitifera*) than to the highly different sets found in anemones such as *N. vectensis, A. pallida* and *A. viridis* (Fig. S8). This exceptionally large and diverse inventory of cnidarian genes associated with symbiosis will help to elucidate molecular models of host-symbiont interactions.

### Large expansion of galaxin genes for coral calcification

**Figure 6:**
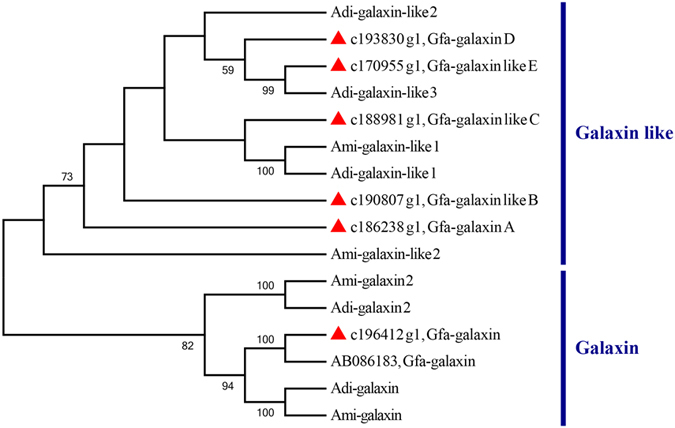
Phylogeny of Galaxin and Galaxin-like proteins. A phylogenetic tree of Galaxin proteins in the corals was created based on the maximum likelihood analysis with the Kimura 2 parameter method. Full–length amino acid sequences were used in this analysis. Percentages of 1,000 bootstrap replicates are indicated next to the tree nodes. Sequences from the present study are marked with solid triangles.

Six unigenes encoding galaxin proteins were identified in the *G. fascicularis* transcriptome based on similarity searching, including one galaxin protein and five galaxin-like proteins. A phylogenetic tree was constructed based on the predicted galaxin and galaxin-like protein sequences. Our results revealed a species-specific pattern in galaxin and galaxin-like proteins between *G. fascicularis* and *Acropora (A. digitifera* and *A. millepora*). No galaxin 2 homolog was found in *G. fascicularis* (Fig. 6). The location of the signal peptides and N-glycosylation sites of galaxin and galaxin-like proteins were predicted using the SignalP 3.0 Serve and NetNGlyc 1.0 Server (http://www.cbs.dtu.dk/services), respectively. The signal peptides of both the galaxin and galaxin-like proteins contained Cys repeat-like regions^26^. Different from galaxin, the galaxin-like precursor proteins of *G. fascicularis* possessed acidic amino acid regions (Asp- and Glu-rich motifs were present in the galaxin-like proteins) that varied widely in sizes ranging from 11 to 135 aa (Fig. S9).

## Discussion

### Molecular basis for coral tolerance to chronic eutrophication

**Figure 7:**
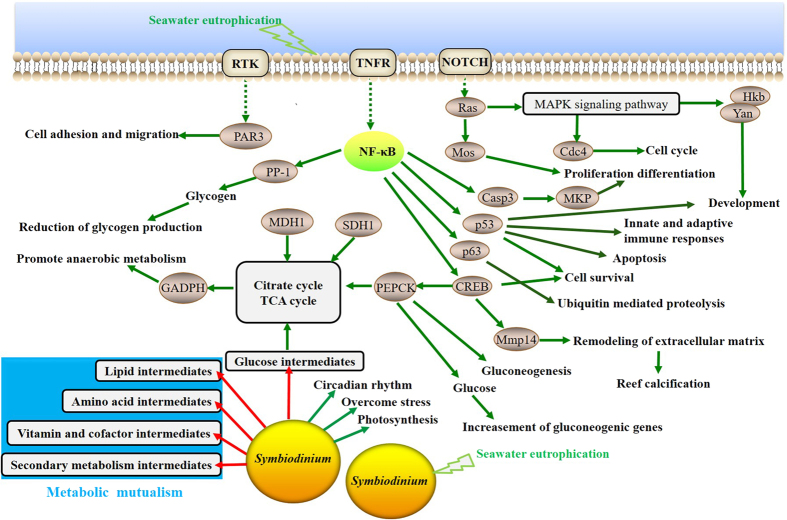
An integrative model summarizing the roles of genes involved in *G. fascicularis* tolerance to coastal chronic eutrophication. The known functional pathways are represented as green lines. The possible functional pathways are represented by red lines.

The water qualities in the eastern and western pools of Hainan were comprehensively analysed in this study. Nitrogen, phosphorus, and chlorophyll-a, which are the most significant factors causing eutrophication, were much more abundant in the western pools. The transcriptome data revealed that 236 genes (FDR corrected *P *<* *0.05) were potentially associated with the chronic eutrophic condition, which demonstrated that this approach provided new insights into the study of the molecular basis of coral eutrophication tolerance. The expression levels of transcription factors and cell signalling molecules associated with tolerance processes changed significantly in coral. The classic transcription factor NF-κB showed remarkable variations in expression levels, which could be used as the ‘smoke-detector’ due to the strong link between NF-κB and different stress signals^27^. Once activated, NF-κB regulates the expression of a large number of downstream processes involved in immunity, apoptosis, cell structuring, adhesion, cell cycle and development, maintenance of energy homeostasis, and response to stress (Fig. 7)^28^. The gene expression pattern of signal transduction in the coral immune response was largely congruent with previous observations in *Acropora palmata*^29^.

The overloading of nutrients favours the growth of algae, aquatic macrophytes and other microbes. Prodigious dissolved oxygen is consumed by the algae and microbes in the respiration and decomposition of organic matter, triggering aquatic hypoxia and cyanotoxin stress in the eutrophication area^30^. We hypothesized that such an environment disfavoured the survival of corals and may result in the stress/immune response of corals. Several receptors showed increased expression in the eutrophic condition, such as DRD1, HTR1, NPFFR2, NPYNR, TNFR1, RTK and Notch. It is very likely that under eutrophication, TNF signalling activated the NF-κB pathway (increased 1.17- and 1.97-fold in eutrophied sites, respectively), which subsequently transduced the signal to its primary target proteins, such as p53, p63 and Casp3 elements. These proteins contributed to governing innate and ‘adaptive-like’ immunity responses, the control of cell survival, differentiation and proliferation. Lesser *et al*. previously detected similar patterns invoked by heat and light stresses in *Montastraea faveolata*^31^. The ubiquitin-mediated proteolysis pathway targets many short-lived cell cycle regulatory proteins, such as cdc14 (1.59-fold), cdk5R1 (1.46-fold) and TOB (1.65-fold)^32^, allowing for quick transitions between cell cycle stages^33^. The expression levels of three calcification genes (Mmp14, SNX10 and SOX9) were also highly variable, changing 2–4 fold in our analysis^26^,^29^.

Maintaining the metabolic balance and energy homeostasis between coral and *Symbiodinium* as the basis for symbiotic systems ensures the survival of individuals. Herein, the KEGG term enrichment analysis (*p* < 0.05) for the coral samples collected from the western pool versus the eastern pool was performed. The eutrophied pool showed increased expression of genes involved in several pathways, including the TCA cycle, glucose metabolism and gluconeogenesis, and decreased expression of the lipid biosynthesis pathway. These genes could also be categorized according to their roles in energy metabolism. Additionally, NF-κB directly or indirectly participates in the regulation of several insulin-related proteins (such as PEPCK, CREB, PP-1, GADPH, KLF3, MDH1 and SDH1) and signalling pathways that are in control of energy demand^34^. Meanwhile, corals have coordinated the expression of genes involved in intermediate transport and exchange between the coral host and *Symbiodinium* during adaptation to eutrophic conditions, enabling them to efficiently reconstruct mutualistic ecology and share metabolism balance^35^. Solute carriers (SLCs) embrace transporters for inorganic substrates or other small organic molecules, *i.e.*, nitrogen, Na^+^, Ca^+^ and amino acid carriers. CYP1C and CYP3A, which belong to the cytochrome P450 family genes, typically catalyse mono-oxygenase reactions involving the metabolism of intermediate substrates^36^. We observed that six SLC genes and these two cytochrome P450 family genes exhibited strikingly different expression levels (fold change > 1.5) in transcriptomic analysis. Our study also indicated that eutrophication induced the expression of several proteins responsible for activating cell self-protection, such as HSP27 (1.03-fold) and opuD (4.01-fold). HSP27 is essential for the response to a wide variety of unfavourable environmental conditions to finely regulate the physiological balance^37^. OpuD is a major osmoprotectant glycine betaine transporter required for early osmotic adjustment^38^. Overall, these results implied the potential for transcriptomic plasticity in the coral and indicated the underlying molecular mechanisms for chronic eutrophication tolerance.

Major *Symbiodinium* shifts of corals occur in severe bleaching events or extreme high temperature episodes^28^. Our study showed that the chronic eutrophied coral colonies did not shift the tendency of *Symbiodinium* C to be the dominant symbiont, but approximately 0.82% (205 genes) of symbiotic *Symbiodinium* genes in the eutrophic state showed differential expression compared to that in the relative pristine state. It is possible that both the coral and *Symbiodinium* have adapted to the eutrophic environment under long-term exposure. Consistently, Daniel *et al*. suggested that *Symbiodinium* is able to adjust gene expression and transcriptional acclimation over long time scales in response to shifting environmental conditions^39^. Our results indicated that eutrophication had significant influences on the photosynthesis (LHCA1, 1.01-fold), circadian rhythm (CSNK1E, 2.07-fold) and stress response (STIP1, 1.83-fold) of symbiotic *Symbiodinium*, in line with the symptoms of eutrophic stress, which include decreased photosynthetic efficiency and increased consumption^4^. Another group was found to be associated with the biosynthesis of secondary metabolites (E1.1.1.122, E3.2.1.21, dxs, PLSC, GPI and MetH) and sucrose metabolism (beta-glucosidase) adjustment for concordance with the coral host gene expression, which may relate to metabolic modulation for the mutualistic continuum of host nutrients/intermediates (Fig. 7). Our results suggested that chronic eutrophication influenced multiple specific biological processes by altering transcription accumulation^40^ and coordinating the expression of two aspects of the coral host and *Symbiodinium* symbionts. However, the mechanisms of global transcriptional changes and their physiological consequences are not yet fully understood.

### Metabolic mutualism between coral and *Symbiodinium* partners

Metabolic mutualism, where each partner produces gene products necessary for mutual survival, reflects the shared metabolites of the partners^41^. Metabolic mutualism is maintained under a plot of reciprocal exploitative interactions simultaneously keeping the contribution of each partner to a minimum while selfishly maximizing their own fitness^42^. Recently, Shinzato and Lin found support for a potentially genetically complementary supply between the coral and *Symbiodinium* genomes in terms of essential amino acids and major photoprotector mycosporine-like amino acids (MMA), forming an interdependent metabolic patchwork for essential nutrient provisioning and photo-protection^19^,^20^,^43^. Many pathways of amino acid synthesis require gene products produced by coral and *Symbiodinium*, while *G. fascicularis* and symbiont seem to lack the expression of genes necessary for histidine, tryptophan, tyrosine and phenylalanine biosynthesis, suggesting yet another source or some yet elusive metabolic patchwork of these amino acids (*i.e*., bacterial endosymbionts)^44^. In our transcriptomics study, we firstly proposed that such a coral host-*Symbiodinium* metabolic mutualism pattern involved vitamin and cofactor, fatty acid and secondary metabolites biosynthesis. Previously, we considered that *Symbiodinium* was a complement machine and provided different resources (*i.e.,* energy source, amino acids, vitamins and cofactors) for the host coral. However, based on the current evidence that little overlap occurs in the biosynthetic genes, the coral and *Symbiodinium* appear to depend on each other for necessary compounds and intermediates in the metabolic processes. These mutualism associations suggest that gene products or intermediates for vitamin and cofactor biosynthesis are shared between coral and *Symbiodinium*. In this regard, interaction intermediates could occur in many aspects of metabolism, and required intermediates or enzymes are somehow available in both systems^23^,^45^, which contributed to create a strong metabolic plasticity and continuity in the face of environmental change. The mutualistic characteristics may extend to the mutual compounds that were redundant in the symbionts or the hosts^46^. Typically, the *Symbiodinium* transcriptome has an ample amount of genes associated with pathways involved in DNA replication, protein processing, transcription, translation, and nutrient provisioning to host^47^,^48^. To our knowledge, the coral host acquires carbon source for energy and nutrition from its symbionts. However, of particular interest to us are the roles of *Symbiodinium* in translocating photosynthetic products as well as the recycling of host metabolic products^35^.

It is worth mentioning that in this study, the coral samples were collected with limited time points and environmental conditions, and it is possible that the completeness of the gene repertoire of the assembled transcriptome is low and that some metabolic genes in the coral or *Symbiodinium* are missing in the dataset. Nevertheless, we discuss the evidence supporting the existence of a mutualistic continuum in *Symbiodinium*-coral interactions and propose a consideration of the evolutionary ecology of these associations^42^. The oligotrophic habitat makes the association between coral host and symbionts driven by metabolic integration desirable^49^. Thus, a metabolic mutualism continuum reflects long-term cooperation and coevolution of the two lineages represented by the coral host and symbiont *Symbiodinium*^50^. There is a more recent hypothesis that vertical transmission of symbionts generally represented a greater number of mutualistic associations, which favours increased metabolic integration that (i) leads to symbiont genome reduction and obligate dependency for a specific host and (ii) genetic uniformity of symbionts and reduction of the number of competitive phenotypes^42^. Most horizontally transmitted symbionts (*i.e*., free-living *Symbiodinium*) are facultative or shift to parasitism, which leads to a reduction in host fitness and steadiness^51^. There is evidence that some species of *Pocillopora* and *Porites* are vertical transmitters and are more resistant to environmental stress^52^. If true, then this could provide novel insights into the potential fitness consequences of the coral-symbiotic mutualistic system, specifically when these associations are exposed to a range of environmental stresses.

### *G. fascicularis* transcriptome characteristics

Here, we reported the first transcriptome generated for a galaxy coral, *G. fascicularis*. In total, we obtained 42.15 Gb of raw sequence data that was assembled into 347,084 unigenes ( ≥ 200 bp). We further characterized 150,771 unigenes based on functional annotation. The BLAST search against the *A. digitifera* and *N. vectensis* datasets identified 24,391 *G. fascicularis* unigenes with hits, a similar number of shared unigenes with other *Anthozoa* species. However, a large amount of sequences could not be matched to the reference genomes. There could be several possibilities for the unmatched reads, such as sequencing and assembling errors, the presence of contaminated sequences and the incompleteness of the reference genomes. In particular, because the coral we used in the transcriptome sequencing was *G. fascicularis*, which taxonomically differs from the reference coral *A. digitifera*^19^, the unblasted unigenes may be derived from the presence of a considerable number of *G. fascicularis*-specific genes or divergently transcribed genes^53^. Because total RNA was isolated from the adult *G. fascicularis* polyps, the transcriptome assembly may contain unigenes from both coral and its zooxanthellae symbionts. Indeed, 25,030 sequences were successfully matched to *Symbiodinium* (clade C and D, C as the dominant symbiont type). In addition, approximately 31,844 and 24,189 unigenes were assumed to be of coral and *Symbiodinium* origin, respectively. All of these data will lay the foundation for further studies on the ecology of interactions between *G. fascicularis* and *Symbiodinium*.

Surveys for major gene families showed that the *G. fascicularis* genome encoded a larger number of transcription factors and signal transduction molecules than did *P. australiensis* and *A. digitifera*. It is argued that as organism complexity increases during evolution, more regulators are required for the regulatory network manifold^54^. The comparisons revealed that biomineralization-related proteins are particularly expanded in *G. fascicularis*. Galaxin, which encodes for a matrix protein suspected to be involved in calcification, is originally identified from the coral *G. fascicularis*^55^. It is hypothesized that the coral galaxin homologs are recruited as biomineralization proteins when Scleractinia diverged from non-biomineralizing taxa during the Triassic^56^ because galaxin-like proteins are also found in non-calcifying taxa outside Cnidaria^50^. Six galaxin coding sequences is a surprisingly high number in *G. fascicularis*, exhibiting a far greater calcific potential^26^. Such enhanced biomineralization and calcification machinery indicates the enhanced reef-building capacity of *G. fascicularis*. Herein, we also identified and comparatively analysed a number of immune-related and symbiosis-associated genes, which revealed significant differences between related coral species. These genes will be of great importance to study the basis of the coral innate immunity network and symbiont recognition and maintenance.

## Conclusions

In this study, the main findings are as follows: (i) Transcriptome-wide gene differential expression analysis of *G. fascicularis* and its endosymbiont *Symbiodinium* was used to determine multiple specific biology processes that play important roles in coral holobiont chronic tolerance to coastal eutrophication. (ii) Metabolic integration was observed in vitamin and cofactor, amino acid, fatty acid, and secondary metabolite biosynthesis between the *G. fascicularis* host and its symbionts at the expression level, providing evidence to support the existence of a metabolic continuum in coral-*Symbiodinium* systems. (iii) Surveys of the *G. fascicularis* transcriptome showed that large sets of transcription factors, cell signal transduction molecules, biomineralization machinery, and galaxin-related proteins were expanded to a comparative scale with other associated coral species. We envision that analysis of the molecular ecology bases of *G. fascicularis* may provide new insights that are critical for the future protection of coral reefs from global marine environmental pollution.

## Materials and Methods

### Ecological setting and experimental design

The western pool of Hainan lies in the Beibu Gulf, which is a semi-enclosed shallow bay bounded by Vietnam, Guangxi, Leizhou Peninsula and Hainan island (Fig. 1A). The Beibu Gulf is subjected to land-based sources of pollutants from industrial, agricultural and aquaculture activities on the northeastern side, which is one of the most industrialized areas in China. Our focus is on three main points of the seawater eutrophication area at the western pool of Hainan, including the Haitou/Yangpu port and an adjacent pool zone, which is fed by two lowland rivers (Zhubi River and Beimen River) that drain agriculture and industry areas of the coastal plain. It is noted that the rivers bring nutrients (especially nitrates and phosphates) from the land to the Gulf. To decipher the transcriptomic landscape of coral tolerance to eutrophication in the western pool, we used the corals originating from the eastern pool of Hainan at the same latitude as the reference to estimate different expression patterns of unigenes. The east coast of Hainan is open to the west-Pacific. Sewage discharge and other forms of pollution have not yet been reported in this area. The two pools we have focused on represent two distinct coral reef water qualities that are similar in depth (1.5–2 m), wave exposure and water delivery^57^. As a prerequisite for this study, we further confirmed by examining the water samples from the eastern and western pools the nutrient concentrations. The results showed that strongly higher concentrations of nutrients were present in western sites due to anthropogenic eutrophication and poor flow in the semi-enclosed pool in the Beibu Gulf. No significant difference in the sea-surface temperature, salinity or water pH was found between the two pools in our investigation. As the environmental condition of the coral reef is a dynamic system, we did not rule out the possibility of other environmental differences, such as minor variability in oxygen or water flow. We used *G. fascicularis* as the coral model in this study because this species lives from shallow water backreefs to deeper fore reef habitats and is highly abundant across the South Sea of China.

### Coral collection, identification and ethical statement

Coral polyps were collected from 12 healthy-looking colonies of *G. fascicularis* living in the eastern and western pools of Hainan island, China, at noon in July 2015 (E1, E2 and E3: samplings from the eastern pool. W1, W2 and W3: samplings from the western pool, see Table S1). The locations of the six sampling sites are shown in Fig. 1B. Two colonies were collected from each sampling site, which yielded 12 samples that were separately prepared for gene expression analysis via RNA-Seq. The polyps were rapidly immersed in 1 mL of TRIzol reagent and frozen in liquid nitrogen. The samples were then stored at −80 °C until processing. The field studies and sample collection activities were necessarily permitted by the Chinese Hainan Government Department of Coral Reefs Protection Provisions. Genomic DNA from the colonies of *G. fascicularis*, which were used in the transcriptome study, was extracted, and the noncoding regions of the mitochondrial genes cytb/nad2 were amplified using PCR with the primers (188-2: 5′-TCCTGTAGAATAGGGTATAC-3′) and (188-R2: 5′ TTTGCCTTTCCGTATCCACCAT-3′)^9^. The cytb/nad2 sequence data from *G. fascicularis* colonies as well as reference sequences were aligned using DNAMAN version 4.0 (Lynnon Biosoft, San Ramon, CA) (Fig. S10). The result presented here confirmed that the 12 corals used in the experiment were from the same lineage and were 100% identical to the sequences of mt-L1 type of *Galaxea* at GenBank (accession number: LC155810 and AB109376)^8^,^10^.

### Water sampling and analysis

Water samples were collected from the western pool, eastern pool and coastal sites of Hainan. Front-reef and Back-reef waters were sampled randomly by boat along a land-sea gradient. Discrete water samples were collected, beginning per coral sampling within 1.0 m of the colony. For dissolved nutrients, near-bottom (1.00 m above bottom) water samples were filtered through 0.45-μm GF/F filters and collected in triplicate in clean 1-L Nalgene bottles. They were frozen in darkness until analysis in the Key Lab of Marine Biogenetic Resources at the Third Institute of Oceanography (TIO). Mercuric chloride (0.03% total volume) was added to the water samples to measure the total dissolved inorganic carbon (DIC) and total alkalinity (TA). For each discrete water sample, DIC was measured using an Apollo SciTech model AS-C3 DIC analyser (Apollo SciTech, DE, USA). TA was measured by open-cell potentiometric titration, and salinity was measured with a Guildline Autosal salinometer^58^. The initial seawater pCO_2_, pH, HCO_3_^−^, CO_3_^2−^, Ω aragonite and Ω calcite were calculated by measurement of TA, DIC and salinity using the CO_2_ SYS program^59^. For chlorophyll a analysis, we collected 800-mL water samples in triplicate of near-bottom water that were filtered (after adding 1 mg of MgCO_3_) through GF/F glass fibre filters. The filters were extracted for 30 min with 10 mL of dimethyl sulfoxide, and we then added 15 mL of 90% acetone at 4 °C overnight. They were measured fluorometrically before and after acidification for the measurement of chlorophyll a and phaeopigment concentrations^60^. The samples were analysed for NH_4_^+^ –N, NO_3_^−^–N, NO_2_^−^–N, PO_4_^3−^ –P and SiO_3_^2−^-Si on a model 7230 Spectrophotometer at the Marine Chemistry Laboratory in TIO. The concentration of dissolved inorganic nitrogen (DIN) is the sum of NO_3_^−^, NO_2_^−^, and NH_4_^+^. After a test for variances within location points, the significance of differences between the location points was assessed using 1-way ANOVA and a t-test via the Tukey method. All statistical tests were performed using IBM SPSS Statistics 23.

### Transcriptome Sequencing and Assembly

Total RNA was extracted from each coral sample using the TRIzol^®^ Reagent RNA Isolation Kit (Invitrogen, Grand Island, NY) following the manufacturer’s protocol. RNA degradation and contamination were detected on 1.2% agarose gels. A total amount of 1.5 μg RNA per sample was used as input material for the RNA sample preparations. mRNA was purified from total RNA using poly-T oligo-attached magnetic beads. Sequencing libraries were generated using NEBNext^®^ Ultra™ RNA Library Prep Kit for Illumina^®^ (NEB, USA) following the manufacturer’s recommendations, and index codes were added to attribute sequences to each sample. The clustering of the index-coded samples was performed on a cBot Cluster Generation System using TruSeq PE Cluster Kit v3-cBot-HS (Illumina) according to the manufacturer’s instructions. After cluster generation, all 12 libraries were sequenced on an Illumina Hiseq 2500 platform at Novogene Biological Information Technology Co., Ltd. (Beijing, China), and paired-end reads were generated.

Raw reads in the fastq format were first processed through in-house perl scripts. Clean reads were obtained by removing reads containing adapter, reads containing poly-N and low quality reads from raw data. At the same time, the Q20, Q30, GC-content and sequence duplication level of the clean data were calculated. Transcriptome *de novo* assembly was accomplished based on the left.fq and the right.fq files from all libraries using Trinity^61^ with min_kmer_cov set to 2 by default and all other parameters set to the default. The obtained sequences were defined as unigenes. Sequence redundancy was removed by searching similar sequences with a minimum similarity cut-off of 95% using CD-HIT-EST. CD-HIT was used for further clustering with a 90% similarity cut-off ref. 62. Each cluster represented by its longest sequence was then combined into one fasta file and used to compare the gene numbers with other organisms.

#### BLAST of the transcriptome with genomes from *Anthozoa* and *Symbiodinium*

To characterize the ‘coral host’ and ‘*Symbiodinium* spp.’ transcriptome assembly, unigenes of the metatranscriptome were first matched using BLASTN (E-value < 1E-10) to genome and transcriptome data from Anthozoa (*A. digitifera* and *N. vectensis*)^19^,^63^. Unigenes not present in Anthozoa were searched against the database containing *Symbiodinium (S. minutum*, clade B and *S. kawagutii*, clade F) genome sequences^18^,^43^. The remaining umatched unigenes were aligned with the NCBI NR protein database using BLASTX (with 90% identity and E-value < 1E-10). The resulting outputs were used to examine the taxonomic assignment^15^,^22^,^53^.

### Gene annotation, gene ontology and metabolic pathway analysis

Gene function was annotated using BLAST with a cutoff E-value of 10^−10^ based on the following seven databases: Nr (NCBI non-redundant protein sequences), Nt (NCBI non-redundant nucleotide sequences), Pfam (Protein family), KOG/COG (Clusters of Orthologous Groups of proteins), Swiss-Prot (a manually annotated and reviewed protein sequence database), KO (KEGG Ortholog database) and GO (Gene Ontology). *A. digitifera* genomic metabolic pathways were predicted using the KEGG Species Server: Acropora digitifera (http://www.kegg.jp/kegg-bin/show_organism?menu_type=pathway_maps&org=adf).

### Quantification and differential expression analysis

Gene expression levels were measured in terms of the fragments per kilobase of exon per million fragments mapped (FPKM) as described by Mortazavi *et al*.^64^. The FPKM for each unigene was calculated by Cufflinks and Cuffdiff^65^. For these analyses, genes with extremely low expression in all libraries (FPKM less than 0.1) were excluded. Differential expression analysis was performed using the DESeq R package (1.10.1)^66^. DESeq provides statistical routines for determining differential expression in digital gene expression data using a model based on the negative binomial distribution. The resulting P values were adjusted using the Benjamini and Hochberg’s approach for controlling the false discovery rate^67^. A corrected *P*-value of 0.05 and log2 (fold change) of ±1 were set as the thresholds for the selection of unigenes with significantly differential expression. We used KOBAS software to test the statistical enrichment of differentially expressed genes in KEGG pathways^68^.

### Phylogenetic analysis

The amino acid sequences of the galaxin family from *G. fascicularis, A. digitifera* and *A. millepora* were used for phylogenetic analysis. Multiple sequence alignment was carried out using ClustalW, and the phylogenetic tree was generated by the Maximum likelihood method using MEGA version 6.0 69. Bootstrap values were calculated from 1,000-replicate analyses.

## Additional Information

**How to cite this article**: Lin, Z. *et al*. Transcriptome profiling of *Galaxea fascicularis* and its endosymbiont *Symbiodinium* reveals chronic eutrophication tolerance pathways and metabolic mutualism between partners. *Sci. Rep.* **7**, 42100; doi: 10.1038/srep42100 (2017).

**Publisher's note:** Springer Nature remains neutral with regard to jurisdictional claims in published maps and institutional affiliations.

## Supplementary Material

Supplementary Additional information
