# Supplementary material for: Transcriptome profiling of Galaxea fascicularis and its endosymbiont Symbiodinium reveals chronic eutrophication tolerance pathways and metabolic mutualism between partners
Source: Sci Rep. 2017 Feb 9;7:42100. doi: 10.1038/srep42100 (PMC5299600; doi:10.1038/srep42100)
Supplement: Supplementary Additional information [file srep42100-s1.doc]

### Additional information

**Transcriptome profiling of Galaxea fascicularis and its endosymbiont *Symbiodinium* reveals chronic eutrophication tolerance pathways and metabolic mutualism between partners**

Zhenyue Lin^1,2 ξ^, Mingliang Chen^2 ξφ^, Xu Dong^3^, Xinqing Zheng^4^, Haining Huang^3^, Xun Xu^1,2^, Jianming Chen^1,2 φ^

^1^ School of Life Sciences, Xiamen University; Fujian Collaborative Innovation Center for Exploitation and Utilization of Marine Biological Resources, Xiamen, Fujian 361005, China

^2^ State Key Laboratory Breeding Base of Marine Genetic Resources; Key Laboratory of Marine Genetic Resources, Third Institute of Oceanography, State Oceanic Administration; Key Laboratory of Marine Genetic Resources of Fujian Province, Xiamen 361005, China

^3^ Laboratory of Marine Chemistry and Environmental Monitoring, Third Institute of Oceanography, State Oceanic Administration, Xiamen, Fujian 361005, China

^4^ Laboratory of Marine Biology and Ecology, Third Institute of Oceanography, State Oceanic Administration, Xiamen, Fujian 361005, China

^ξ^ These authors contributed equally to this work.

^φ^ Corresponding authors

Dr. Jianming Chen

Tel: 86-592-2195086

Email: [chenjianming@tio.org.cn](mailto:chenjianming@tio.org.cn)

Dr. Mingliang Chen

Tel: 86-592-2195393

Email: [mlchen_gg@tio.org.cn](mailto:mlchen_gg@tio.org.cn)

**Supplementary Fig S1-S8**

**
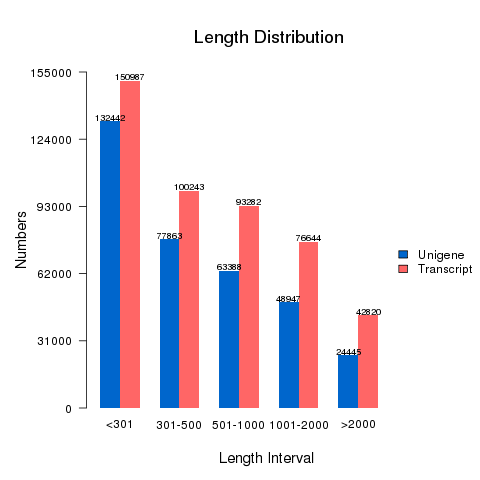
**

**Fig S1.** Length distribution of assembled transcripts and unigenes in *G. fascicularis* polyps. The horizontal axis is the length of transcripts and unigenes, and the vertical axis is the number of transcripts and unigenes.


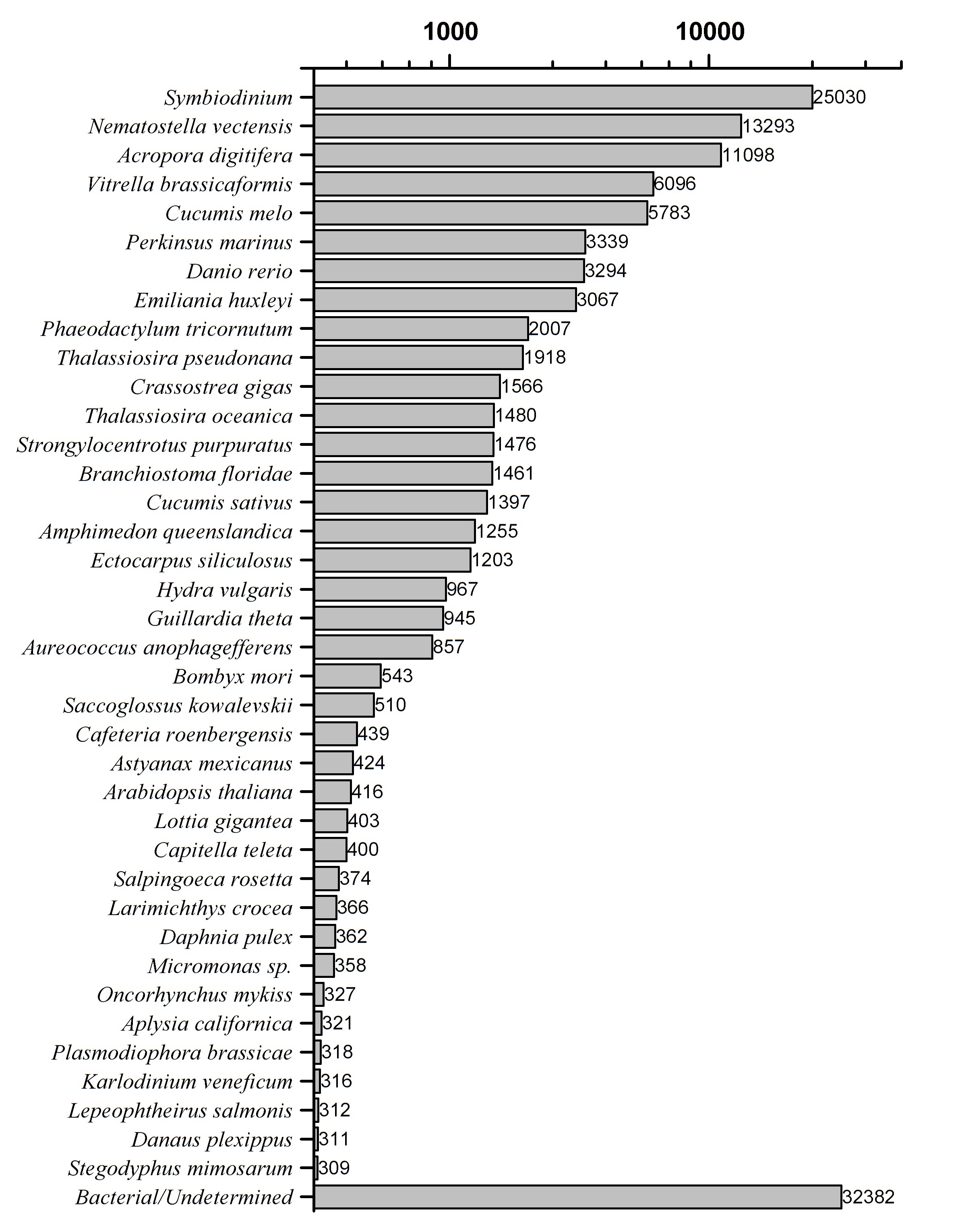


**Fig S2.** Species distribution of top BLAST results. The histogram shows the species distribution of unigenes from blastn (E value<1e-10) to genome data from *A. digitifera*, *N. vectensis* and *Symbiodinium*, as well as the top blastx results against the NR protein database with a cutoff E value<1e-10.


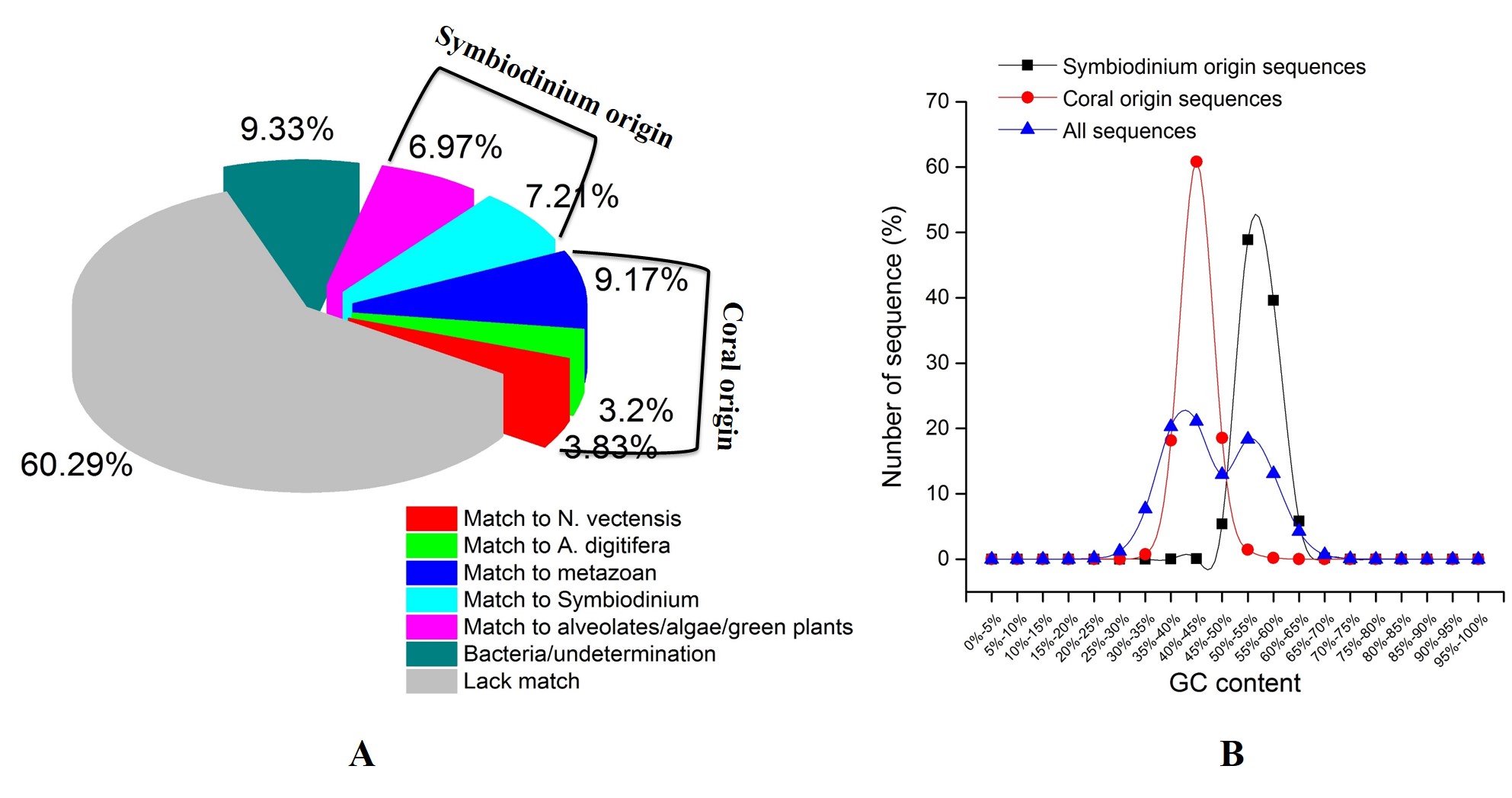


**Fig S3.** (A) Percentage of coral (*G. fascicularis*) and *Symbiodinium* origin of unigenes in the transcriptome. (B) Distribution of GC contents of the assembled sequences.


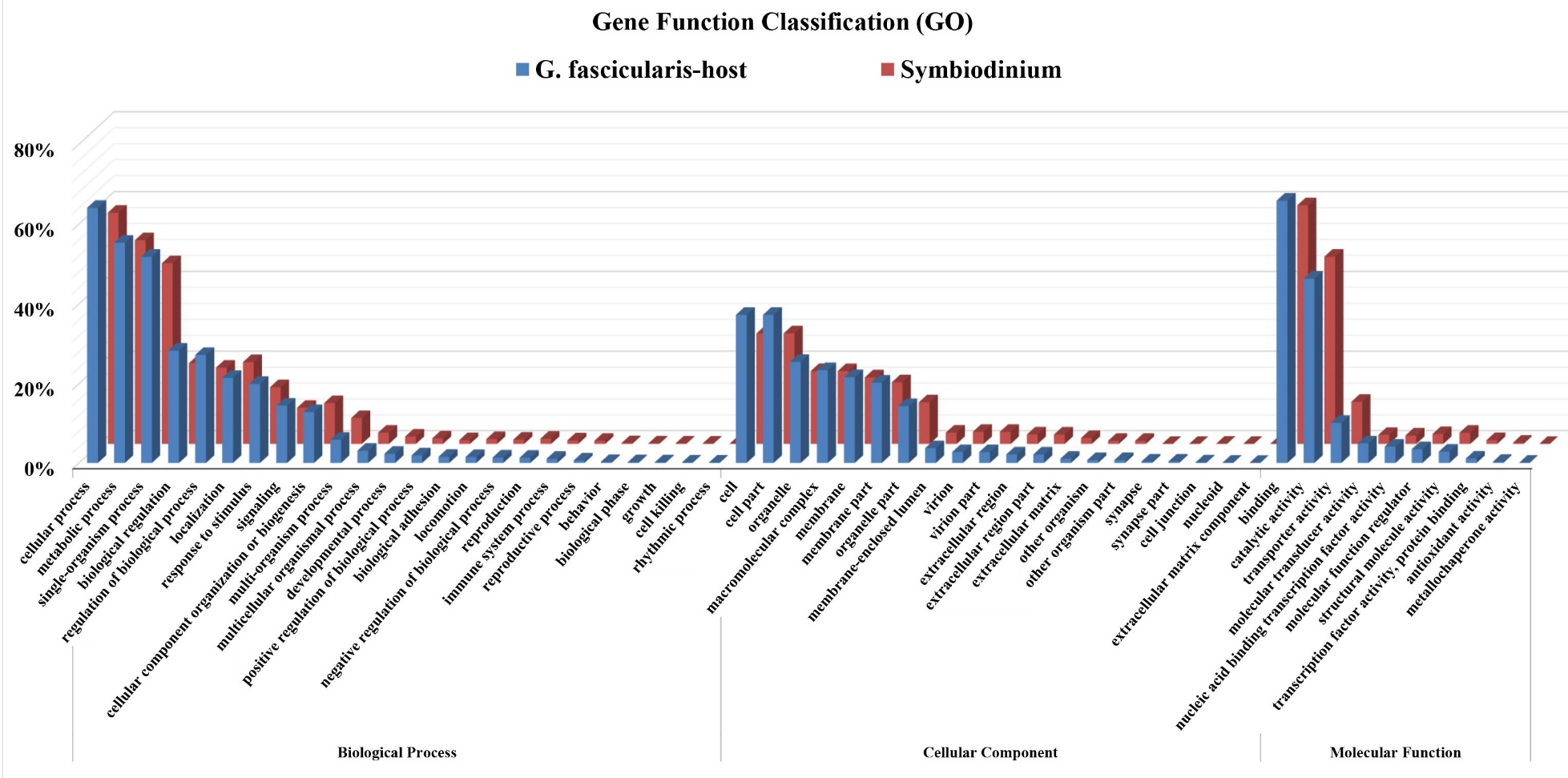


**Fig S4.** Large-scale comparison of GO **categorization** from the transcriptome component of *G. fascicularis*-host and *Symbiodinium*. The enrichment analysis (y-axis) is expressed as the percentage of sequences of *G. fascicularis* and its *Symbiodinium* symbiont transcriptome sets, respectively.


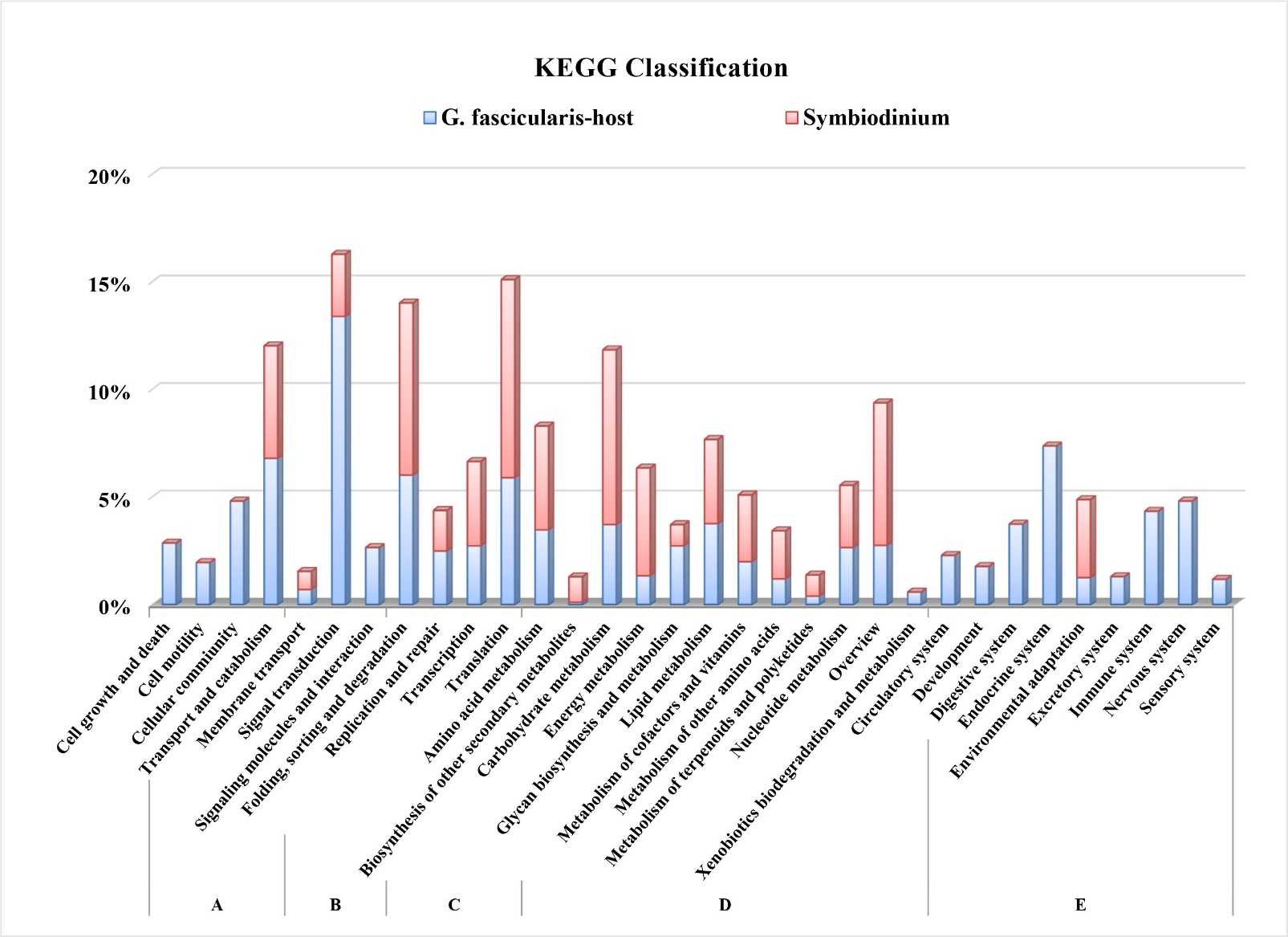


**Fig S5.** Large-scale comparison of KEGG categories assigned to *G. fascicularis* and its *Symbiodinium* symbiont transcriptomes. The enrichment analysis (y-axis) is expressed as the percentage of sequences at *G. fascicularis* and its *Symbiodinium* symbiont transcriptome sets, respectively.

**
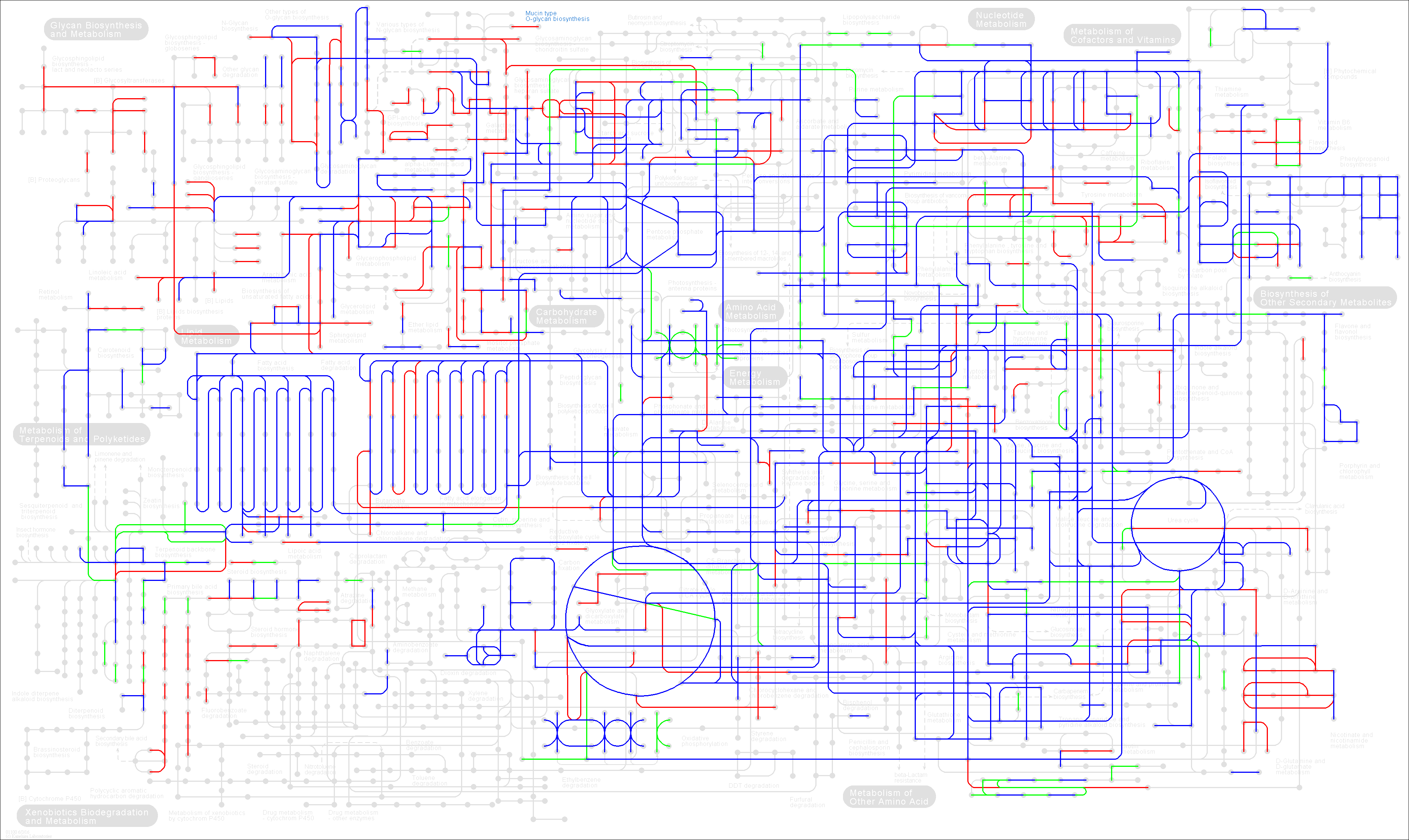
**

**Fig S6.** Basal metabolism pathways of *G. fascicularis* holobiont. Grey lines indicate genes absent in both *Symbiodinium* and coral (*G. fascicularis*) transcriptome. Blue lines indicate genes present in both the *Symbiodinium* and *G. fascicularis* transcriptome. Red lines indicate genes only present in the *G. fascicularis* transcriptome. Green lines indicate genes only present in the *Symbiodinium* transcriptome.


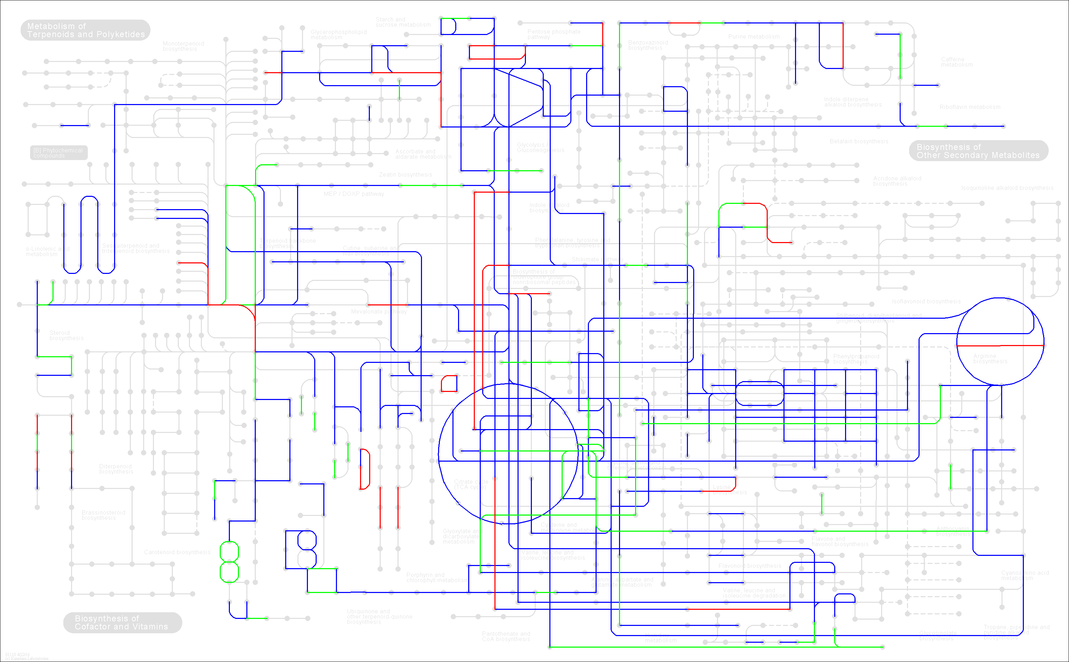


**Fig S7.** *G. fascicularis* holobiont biosynthesis of secondary metabolites. Grey lines indicate genes absent in both the *Symbiodinium* and coral (*G. fascicularis*) transcriptome. Blue lines indicate genes present in both the *Symbiodinium* and *G. fascicularis* transcriptome. Red lines indicate genes only present in the *G. fascicularis* transcriptome. Green lines indicate genes only present in the *Symbiodinium* transcriptome.


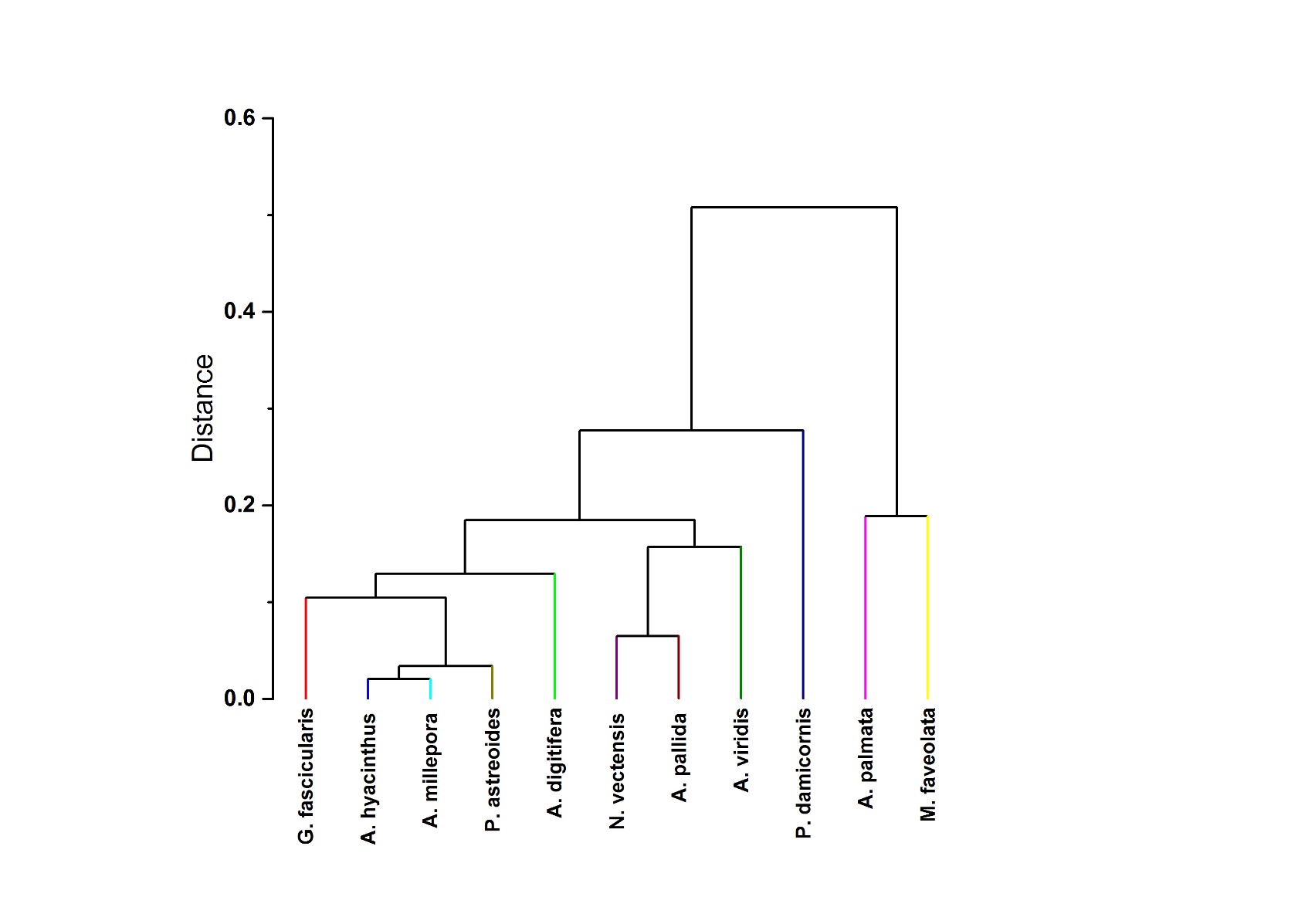


**Fig S8.** Hierarchical clustering of candidate genes associated with symbiosis from *G. fascicularis* and other cnidarian sequences.


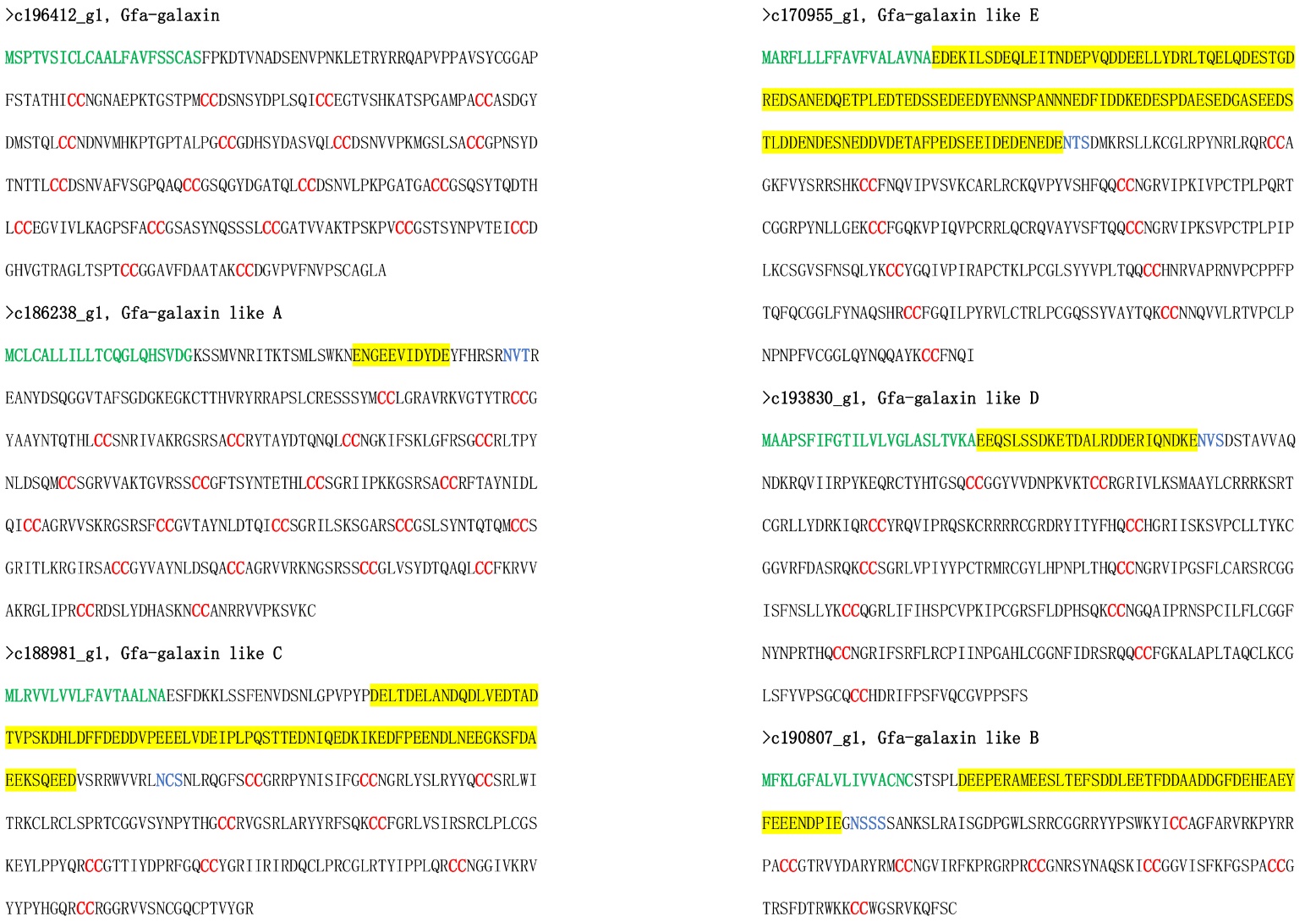


**Fig S9.** Inferred protein sequences of galaxin and the galaxin-like molecules from *G. fascicularis*. Galaxin differs from the galaxin-like molecules in that it lacks an acidic domain. The signal peptide is marked in green, the acidic domain in yellow, and the Cys repeats in red. Potential N-linked glycosylation sites in galaxin and galaxin-like are shown in blue.

**Fig S10.** Sequence alignment of the noncoding region mitochondrial genes (cytb/nad2) of *Galaxea*, which used in this study as well as reference sequences obtained from NCBI.

**Supplementary Table S1-S11**

**Table S1. Summary of sequencing analysis.**

| **Sample** | **Location** | **Raw reads** | **Clean reads** | **Clean bases** | | **Error(%)** | **Q20(%)** | **Q30(%)** | **GC(%)** | **SRA accession no.** | |
| --- | --- | --- | --- | --- | --- | --- | --- | --- | --- | --- | --- |
| **W1（2 colonies）** | E108°90′,N19°48′ | 50,577,978 | 48,954,684 | 7.34G | 0.01 | | 96.74 | 92.32 | 44.98 | | SRR3575053 |
| **W2（2 colonies）** | E108°88′,N19°47′ | 49,596,636 | 48,080,484 | 7.21G | 0.01 | | 96.80 | 92.39 | 48.15 | | SRR3575054 |
| **W3（2 colonies）** | E108°96′,N19°55′ | 41,912,492 | 40,672,212 | 6.10G | 0.01 | | 96.81 | 92.40 | 48.06 | | SRR3575055 |
| **E1（2 colonies）** | E110°63′,N19°20′ | 48,893,708 | 47,461,454 | 7.12G | 0.01 | | 96.89 | 92.54 | 51.14 | | SRR3575056 |
| **E2（2 colonies）** | E110°65′,N19°25′ | 46,799,940 | 45,371,376 | 6.81G | 0.01 | | 96.94 | 92.67 | 50.44 | | SRR3575057 |
| **E3（2 colonies）** | E110°69′,N19°31′ | 51,931,058 | 50,481,462 | 7.57G | 0.01 | | 97.02 | 97.02 | 48.40 | | SRR3575058 |
| **Total（12 colonies）** |  | 289,711,812 | 281,021,672 | 42.15G |  | |  |  |  | |  |

E1, E2 and E3: sampling from east pool.

W1, W2 and W3: sampling from west pool.

Q20: The percentage of bases with a Phred value > 20.

Q30: The percentage of bases with a Phred value > 30.

**Table S2. **BLAST analysis of non-redundant unigenes against public databases.****

| **The public databases** | **Number of Unigenes** | **Percentage (%)** |
| --- | --- | --- |
| Annotated in NR | 62094 | 17.89% |
| Annotated in NT | 26666 | 7.68% |
| Annotated in KO | 42359 | 12.20% |
| Annotated in SwissProt | 82497 | 23.77% |
| Annotated in PFAM | 116889 | 33.68% |
| Annotated in GO | 38896 | 11.21% |
| Annotated in KOG | 60451 | 17.42% |
| Annotated in all Databases | 3300 | 0.95% |
| Annotated in at least one Database | 150771 | 43.44% |
| Total Unigenes | 347085 | 100.00% |

**Table S3.** Transcriptome statistics for *G. fascicularis* and its *Symbiodinium* symbionts.

|  | *G. fascicularis* | *Symbiodinium* |
| --- | --- | --- |
| number of unigenes | 56,235 | 49,219 |
| Total assembly size (Mb) | 59.05 | 53.72 |
| N50 | 2,735 | 1,893 |
| GC% | 42.96% | 54.91% |
| Number of Go term | 8,460 | 17,177 |
| Number of KO | 6,752 | 9,307 |

**Table S4.** Genes found in both *G. fascicularis* populations with statistically significant changes in expression from the east and west pools. Common names and E-values are for the top BLAST hit to the SwissProt database.

| **gene_id** | **FPKM** | | **log2FoldChange** | **pval** | **NR Evalue** | **KO Name** | **KO Description** |
| --- | --- | --- | --- | --- | --- | --- | --- |
|  | **East pool** | **West pool** |  |  |  |  |  |
| c212363_g2 | 184.72 | 34.20 | 2.43 | 0.00 | 0 | ADAM9 | disintegrin and metalloproteinase domain-containing protein 9 |
| c209620_g1 | 251.47 | 546.81 | -1.12 | 0.04 | 0 | ALOX5 | arachidonate 5-lipoxygenase |
| c209885_g1 | 176.47 | 36.28 | 2.28 | 0.04 | 0 | ALOX5 | arachidonate 5-lipoxygenase |
| c198201_g1 | 65.59 | 14.95 | 2.13 | 0.03 | 0 | AOC1, ABP1 | diamine oxidase |
| c206331_g2 | 211.20 | 85.98 | 1.30 | 0.03 | 3.06E-99 | BAAT | bile acid-CoA:amino acid N-acyltransferase |
| c183670_g1 | 12.82 | 44.26 | -1.79 | 0.05 | 2.11E-25 | BARHL | BarH-like |
| c199315_g1 | 1153.16 | 2824.51 | -1.29 | 0.05 | 0 | BHMT | betaine-homocysteine S-methyltransferase |
| c210559_g1 | 131.99 | 416.01 | -1.66 | 0.01 | 7.49E-67 | CASP3 | caspase 3 |
| c198416_g1 | 1432.45 | 4301.91 | -1.59 | 0.02 | 0 | CDC14 | cell division cycle 14 |
| c189255_g1 | 399.73 | 1096.25 | -1.46 | 0.01 | 3.11E-79 | CDK5R1 | cyclin-dependent kinase 5 activator 1 |
| c206663_g1 | 1819.57 | 13867.14 | -2.93 | 0.04 | 0 | clpB | ATP-dependent Clp protease ATP-binding subunit ClpB |
| c200545_g1 | 33.35 | 101.72 | -1.61 | 0.03 | 0 | CPD | carboxypeptidase D |
| c197488_g1 | 667.52 | 2130.68 | -1.67 | 0.02 | 1.39E-66 | CREB3 | cyclic AMP-responsive element-binding protein 3 |
| c211150_g1 | 2740.51 | 8681.39 | -1.66 | 0.00 | 0 | CRY | cryptochrome |
| c197982_g1 | 169.54 | 462.46 | -1.45 | 0.02 | 4.61E-28 | CSTA_B | cystatin-A/B |
| c192878_g1 | 63.18 | 330.44 | -2.39 | 0.01 | 4.30E-117 | CTRB | chymotrypsin |
| c208187_g1 | 271.57 | 1330.99 | -2.29 | 0.00 | 1.67E-130 | CYP1C | cytochrome P450, family 1, subfamily C |
| c208001_g1 | 46.20 | 128.52 | -1.48 | 0.05 | 2.22E-180 | CYP3A | cytochrome P450, family 3, subfamily A |
| c183098_g1 | 333.65 | 1070.35 | -1.68 | 0.02 | 4.56E-33 | DBP | albumin D box-binding protein |
| c199595_g1 | 4.11 | 29.94 | -2.87 | 0.01 | 0 | deoA, TYMP | thymidine phosphorylase |
| c206716_g1 | 158.78 | 457.76 | -1.53 | 0.02 | 6.50E-125 | DHDDS, RER2, SRT1 | ditrans,polycis-polyprenyl diphosphate synthase |
| c203854_g2 | 400.61 | 860.51 | -1.10 | 0.04 | 2.86E-179 | DMRT4_5, DMRTA | doublesex- and mab-3-related transcription factor 4/5 |
| c201143_g1 | 1154.22 | 2430.89 | -1.07 | 0.04 | 1.14E-98 | DNAJB4 | DnaJ homolog subfamily B member 4 |
| c200007_g1 | 583.58 | 1273.17 | -1.13 | 0.05 | 1.06E-131 | DRD1 | dopamine receptor D1 |
| c198722_g1 | 1469.36 | 4160.89 | -1.50 | 0.00 | 3.44E-134 | DUSP, MKP | dual specificity MAP kinase phosphatase |
| c210948_g1 | 1600.04 | 4456.26 | -1.48 | 0.01 | 8.54E-66 | DUSP, MKP | dual specificity MAP kinase phosphatase |
| c192312_g1 | 828.90 | 1853.09 | -1.16 | 0.03 | 1.19E-133 | DUSP, MKP | dual specificity MAP kinase phosphatase |
| c208353_g1 | 511.50 | 1534.29 | -1.58 | 0.01 | 6.85E-122 | E3.4.24.21 | astacin |
| c201873_g4 | 83.55 | 215.25 | -1.37 | 0.02 | 2.75E-99 | E3.4.24.21 | astacin |
| c201526_g1 | 7416.28 | 62143.88 | -3.07 | 0.03 | 0 | E4.1.1.32, pckA, PEPCK | phosphoenolpyruvate carboxykinase (GTP) |
| c195852_g2 | 3465.32 | 10209.17 | -1.56 | 0.02 | 0 | EIF4A3, FAL1 | ATP-dependent RNA helicase |
| c189750_g1 | 4513.42 | 10840.55 | -1.26 | 0.02 | 1.89E-26 | ETV6_7, yan | ETS translocation variant 6/7 |
| c210220_g1 | 733.22 | 259.70 | 1.50 | 0.02 | 1.70E-50 | FER, TYK3 | tyrosine-protein kinase Fer |
| c190684_g1 | 114.85 | 480.88 | -2.07 | 0.04 | 5.47E-159 | FOXB | forkhead box protein B |
| c194526_g1 | 228.74 | 557.55 | -1.29 | 0.04 | 3.20E-177 | galE, GALE | UDP-glucose 4-epimerase |
| c203215_g1 | 4728.89 | 11547.42 | -1.29 | 0.04 | 2.20E-179 | GAPDH, gapA | glyceraldehyde 3-phosphate dehydrogenase |
| c199744_g1 | 175.05 | 451.15 | -1.37 | 0.01 | 5.65E-38 | GBX | homeobox protein GBX |
| c211663_g1 | 240.02 | 661.56 | -1.46 | 0.03 | 0 | GLB1, ELNR1 | beta-galactosidase |
| c190507_g1 | 151.08 | 343.46 | -1.18 | 0.04 | 4.02E-24 | HAND1 | heart-and neural crest derivatives-expressed protein 1 |
| c191721_g1 | 707.75 | 3065.76 | -2.11 | 0.00 | 1.13E-36 | HES1 | hairy and enhancer of split 1 |
| c204262_g1 | 936.06 | 3956.58 | -2.08 | 0.00 | 2.20E-21 | HEY | hairy and enhancer of split related with YRPW motif |
| c189110_g1 | 111.04 | 334.74 | -1.59 | 0.01 | 2.34E-39 | HKB | huckebein |
| c202896_g1 | 17.59 | 63.28 | -1.85 | 0.03 | 1.30E-25 | HLX1 | homeobox protein HLX1 |
| c203855_g1 | 53.71 | 186.90 | -1.80 | 0.04 | 7.42E-78 | HSD17B1 | 17beta-estradiol 17-dehydrogenase |
| c210430_g1 | 2435.87 | 4984.89 | -1.03 | 0.03 | 0 | HSPBAP1 | HSPB1-associated protein 1 |
| c209991_g1 | 27.06 | 88.60 | -1.71 | 0.05 | 3.35E-103 | HTR1 | 5-hydroxytryptamine receptor 1 |
| c194010_g1 | 14.22 | 43.45 | -1.61 | 0.05 | 5.00E-86 | IFI30, GILT | interferon, gamma-inducible protein 30 |
| c199769_g1 | 207.71 | 762.50 | -1.88 | 0.00 | 1.11E-45 | IRF2 | interferon regulatory factor 2 |
| c193738_g1 | 196.10 | 524.59 | -1.42 | 0.03 | 1.62E-40 | IRF2 | interferon regulatory factor 2 |
| c193386_g1 | 102.03 | 30.27 | 1.75 | 0.04 | 1.88E-44 | K14165 | atypical dual specificity phosphatase |
| c204033_g1 | 57.05 | 201.25 | -1.82 | 0.04 | 0 | KCNC1 | potassium voltage-gated channel Shaw-related subfamily C member 1 |
| c208007_g1 | 46.73 | 8.18 | 2.51 | 0.01 | 7.34E-60 | KDR, VEGFR2 | kinase insert domain protein receptor |
| c203609_g1 | 1603.58 | 4332.69 | -1.43 | 0.01 | 1.00E-59 | KLF10_11, TIEG | krueppel-like factor 10/11 |
| c199434_g1 | 380.67 | 1062.62 | -1.48 | 0.01 | 1.61E-51 | KLF3 | krueppel-like factor 3 |
| c205051_g2 | 2479.89 | 16922.32 | -2.77 | 0.00 | 0 | KLHL1_4_5 | kelch-like protein 1/4/5 |
| c199453_g2 | 1.78 | 19.31 | -3.44 | 0.05 | 6.56E-36 | KRAB | KRAB domain-containing zinc finger protein |
| c209797_g1 | 1260.77 | 2525.88 | -1.00 | 0.04 | 6.75E-42 | KRAB | KRAB domain-containing zinc finger protein |
| c207577_g1 | 92.54 | 28.52 | 1.70 | 0.03 | 7.60E-155 | LPCAT1_2 | lysophosphatidylcholine acyltransferase / lyso-PAF acetyltransferase |
| c200529_g1 | 1019.14 | 13081.58 | -3.68 | 0.00 | 0 | map | methionyl aminopeptidase |
| c209361_g1 | 1638.71 | 4974.46 | -1.60 | 0.01 | 9.62E-136 | MAPK4_6 | mitogen-activated protein kinase 4/6 |
| c197263_g1 | 2676.02 | 8828.47 | -1.72 | 0.04 | 4.57E-172 | MDH1 | malate dehydrogenase |
| c204152_g2 | 182.91 | 483.20 | -1.40 | 0.03 | 3.87E-69 | MEP1B | meprin B |
| c200667_g3 | 1541.34 | 5520.19 | -1.84 | 0.04 | 0 | metK | S-adenosylmethionine synthetase |
| c205697_g2 | 2781.76 | 6277.87 | -1.17 | 0.03 | 1.14E-128 | MMP14 | matrix metalloproteinase-14 (membrane-inserted) |
| c203081_g1 | 2196.22 | 8558.15 | -1.96 | 0.00 | 2.76E-98 | MOS | proto-oncogene serine/threonine-protein kinase mos |
| c199463_g1 | 957.13 | 286.36 | 1.74 | 0.01 | 5.04E-71 | MSI | RNA-binding protein Musashi |
| c212418_g1 | 527.68 | 1443.61 | -1.45 | 0.01 | 1.34E-82 | MTX | metaxin |
| c204297_g2 | 285.04 | 1476.88 | -2.37 | 0.00 | 1.32E-53 | MXD, MAD | MAX dimerization protein |
| c210817_g1 | 52.67 | 172.66 | -1.71 | 0.05 | 0 | NCAPG2, LUZP5 | condensin-2 complex subunit G2 |
| c203310_g1 | 2272.55 | 7030.08 | -1.63 | 0.01 | 9.55E-15 | NDUFA4 | NADH dehydrogenase (ubiquinone) 1 alpha subcomplex subunit 4 |
| c199573_g1 | 2163.24 | 8474.99 | -1.97 | 0.00 | 5.47E-84 | NFKB1 | nuclear factor NF-kappa-B p105 subunit |
| c210986_g1 | 181.78 | 497.33 | -1.45 | 0.04 | 1.61E-50 | NID | nidogen (entactin) |
| c212335_g1 | 66.19 | 439.13 | -2.73 | 0.01 | 3.20E-143 | NOTCH | Notch |
| c206794_g1 | 442.09 | 1199.00 | -1.44 | 0.02 | 2.33E-66 | NOTCH | Notch |
| c212515_g1 | 862.35 | 2468.91 | -1.52 | 0.01 | 3.15E-113 | NPAS1_3 | neuronal PAS domain-containing protein 1/3 |
| c208897_g1 | 191.19 | 391.39 | -1.03 | 0.05 | 1.03E-81 | NPFFR2 | neuropeptide FF receptor 2 |
| c205669_g1 | 79.66 | 177.26 | -1.15 | 0.04 | 7.56E-72 | NPYNR | neuropeptide Y receptor, invertebrate |
| c186227_g1 | 105.24 | 319.27 | -1.60 | 0.01 | 0 | NR2F1, TFCOUP1 | COUP transcription factor 1 |
| c213221_g2 | 375.75 | 153.51 | 1.29 | 0.04 | 0 | NUG2, GNL2 | nuclear GTP-binding protein |
| c197770_g1 | 186.87 | 7.84 | 4.01 | 0.03 | 6.46E-175 | opuD, betL | glycine betaine transporter |
| c208526_g1 | 328.81 | 675.41 | -1.04 | 0.04 | 2.81E-89 | OTX2 | homeobox protein OTX2 |
| c210431_g1 | 727.99 | 1789.60 | -1.30 | 0.03 | 0 | PARD3 | partitioning defective protein 3 |
| c211946_g1 | 138.63 | 56.61 | 1.29 | 0.04 | 0 | PIK3C2 | phosphatidylinositol-4-phosphate 3-kinase |
| c201287_g1 | 16.97 | 71.19 | -2.07 | 0.04 | 6.86E-90 | POU1F, PIT1 | POU domain transcription factor, class 1 |
| c207183_g1 | 33.04 | 495.22 | -3.91 | 0.00 | 1.61E-33 | PPP1R3 | protein phosphatase 1 regulatory subunit 3A/B/C/D/E |
| c196853_g1 | 1718.42 | 6641.10 | -1.95 | 0.01 | 9.55E-48 | PPP1R3 | protein phosphatase 1 regulatory subunit 3A/B/C/D/E |
| c208332_g2 | 119.29 | 38.69 | 1.62 | 0.05 | 3.33E-155 | RABGGTA | geranylgeranyl transferase type-2 subunit alpha |
| c200216_g1 | 535.62 | 1201.31 | -1.17 | 0.05 | 4.06E-51 | RERG | Ras-related and estrogen-regulated growth inhibitor |
| c212848_g1 | 394.42 | 156.19 | 1.34 | 0.03 | 1.43E-76 | RET | proto-oncogene tyrosine-protein kinase Ret |
| c190611_g1 | 50.70 | 178.92 | -1.82 | 0.01 | 1.22E-78 | RHOB | Ras homolog gene family, member B |
| c195363_g1 | 89.42 | 338.37 | -1.92 | 0.01 | 3.87E-97 | RNF144 | E3 ubiquitin-protein ligase RNF144 |
| c216027_g1 | 0 | 7.43 | -5.37 | 0.04 | 0 | RP-S14e, RPS14 | small subunit ribosomal protein S14e |
| c187997_g1 | 1415.16 | 3761.46 | -1.41 | 0.01 | 1.22E-153 | RUNX1, AML1 | runt-related transcription factor 1 |
| c244990_g1 | 0 | 9.70 | -5.58 | 0.03 | 0 | SDA1, SDAD1 | protein SDA1 |
| c206877_g1 | 531.57 | 1986.89 | -1.90 | 0.04 | 5.07E-34 | SDHD, SDH4 | succinate dehydrogenase (ubiquinone) membrane anchor subunit |
| c201695_g2 | 973.54 | 3531.63 | -1.86 | 0.01 | 3.49E-163 | SLC25A10, DIC | solute carrier family 25 (mitochondrial dicarboxylate transporter), member 10 |
| c205860_g2 | 401.02 | 996.19 | -1.31 | 0.02 | 2.07E-113 | SLC25A33_36, RIM2 | solute carrier family 25, member 33/36 |
| c208423_g1 | 175.01 | 1156.47 | -2.72 | 0.00 | 0 | SLC32A, VGAT | solute carrier family 32 (vesicular inhibitory amino acid transporter) |
| c205553_g2 | 4682.37 | 15329.13 | -1.71 | 0.00 | 0 | SLC34A, NPT, nptA | solute carrier family 34 (sodium-dependent phosphate cotransporter) |
| c212237_g1 | 224.31 | 606.04 | -1.43 | 0.03 | 1.31E-121 | SLC38A10 | solute carrier family 38 (sodium-coupled neutral amino acid transporter), member 10 |
| c207074_g1 | 2955.56 | 7631.67 | -1.37 | 0.03 | 0 | SLC5A8_12, SMCT | solute carrier family 5 (sodium-coupled monocarboxylate transporter), member 8/12 |
| c189238_g1 | 580.50 | 1494.74 | -1.36 | 0.02 | 2.95E-130 | SNAI2, SLUG | snail 2 |
| c202827_g1 | 291.15 | 801.68 | -1.46 | 0.02 | 1.02E-30 | SNX10_11 | sorting nexin-10/11 |
| c196064_g1 | 1254.47 | 2864.19 | -1.19 | 0.02 | 0 | SOX9 | transcription factor SOX9 (SOX group E) |
| c195311_g1 | 1606.63 | 4306.27 | -1.42 | 0.01 | 1.93E-159 | SPSB1_4, SSB1, SSB4 | SPRY domain-containing SOCS box protein 1/4 |
| c202957_g1 | 3075.71 | 15469.04 | -2.33 | 0.00 | 1.53E-81 | SPSB3, SSB3 | SPRY domain-containing SOCS box protein 3 |
| c208970_g1 | 2401.26 | 8254.87 | -1.78 | 0.00 | 2.27E-81 | SPSB3, SSB3 | SPRY domain-containing SOCS box protein 3 |
| c211615_g1 | 1297.37 | 2932.76 | -1.18 | 0.05 | 2.65E-158 | SULF | extracellular sulfatase Sulf |
| c205674_g2 | 70.09 | 20.62 | 1.77 | 0.04 | 4.24E-43 | TC.MATE, SLC47A, norM, mdtK, dinF | multidrug resistance protein, MATE family |
| c204893_g1 | 709.66 | 2226.91 | -1.65 | 0.02 | 0 | TEKT1 | tektin-1 |
| c189579_g1 | 3627.47 | 11345.10 | -1.65 | 0.00 | 1.52E-34 | TOB | protein Tob/BTG |
| c170930_g1 | 2635.99 | 7135.31 | -1.44 | 0.01 | 4.04E-41 | TOB | protein Tob/BTG |
| c195862_g1 | 98.56 | 357.42 | -1.86 | 0.00 | 8.85E-46 | TP53INP1 | tumor protein p53-inducible nuclear protein 1 |
| c206995_g1 | 22.16 | 100.89 | -2.19 | 0.00 | 3.21E-96 | TP63 | tumor protein p63 |
| c209504_g3 | 281.72 | 633.42 | -1.17 | 0.04 | 4.62E-142 | TRAF4 | TNF receptor-associated factor 4 |
| c204821_g1 | 98.46 | 407.90 | -2.05 | 0.00 | 2.29E-45 | TRIM71 | tripartite motif-containing protein 71 |
| c211462_g1 | 1047.73 | 2741.30 | -1.39 | 0.03 | 0 | XPO1, CRM1 | exportin-1 |

**Table S5.** Top *Symbiodinium* species distributions for the highly abundant ITS2 gene in coral samples.

**Table S6.** Genes found in both *Symbiodinium* with statistically significant changes in expression from the east and west pools. Common names and E-values are for the top BLAST hit to the NR database.

| **Gene id** | **FPKM** | | **log2FoldChange** | **pval** | **NR Evalue** | **KO Name** | **KO Description** |
| --- | --- | --- | --- | --- | --- | --- | --- |
|  | **East pool** | **West pool** |  |  |  |  |  |
| c88929_g1 | 176.50 | 71.86 | 1.30 | 0.01 | 3.53E-103 | ABA3 | molybdenum cofactor sulfurtransferase |
| c119774_g1 | 14.95 | 1.38 | 3.43 | 0.01 | 1.08E-64 | ABC.X2.P | putative ABC transport system permease protein |
| c150899_g1 | 547.91 | 251.07 | 1.13 | 0.00 | 0 | AQR | intron-binding protein aquarius |
| c31087_g1 | 42.12 | 114.01 | -1.44 | 0.04 | 4.29E-148 | CDKAL1 | threonylcarbamoyladenosine tRNA methylthiotransferase CDKAL1 |
| c138173_g1 | 80.14 | 165.46 | -1.05 | 0.03 | 2.57E-122 | CPK | calcium-dependent protein kinase |
| c144047_g1 | 58.75 | 14.02 | 2.07 | 0.00 | 2.97E-80 | CSNK1E | casein kinase 1, epsilon |
| c45528_g1 | 92.60 | 45.38 | 1.03 | 0.01 | 5.73E-40 | DHRS7 | dehydrogenase/reductase SDR family member 7 |
| c113270_g1 | 58.57 | 28.49 | 1.04 | 0.04 | 1.06E-121 | DMC1 | meiotic recombination protein DMC1 |
| c19861_g1 | 54.12 | 18.87 | 1.52 | 0.01 | 2.72E-117 | DMC1 | meiotic recombination protein DMC1 |
| c265565_g1 | 83.05 | 34.98 | 1.25 | 0.01 | 3.75E-66 | dnaJ | molecular chaperone DnaJ |
| c170857_g1 | 310.33 | 153.63 | 1.01 | 0.02 | 2.94E-14 | DNAJB11 | DnaJ homolog subfamily B member 11 |
| c54809_g1 | 48.01 | 135.76 | -1.50 | 0.02 | 8.79E-37 | DPO1, polA | DNA polymerase I |
| c149660_g2 | 416.09 | 150.04 | 1.47 | 0.00 | 0 | dxs | 1-deoxy-D-xylulose-5-phosphate synthase |
| c155905_g1 | 87.13 | 43.56 | 1.00 | 0.04 | 2.06E-83 | E1.1.1.- | -- |
| c144467_g1 | 50.40 | 124.38 | -1.30 | 0.01 | 2.15E-66 | E1.1.1.122 | D-threo-aldose 1-dehydrogenase |
| c125316_g1 | 87.24 | 42.55 | 1.04 | 0.03 | 2.76E-73 | E1.11.1.11 | L-ascorbate peroxidase |
| c37890_g1 | 132.97 | 298.78 | -1.17 | 0.00 | 4.81E-159 | E2.3.1.88 | peptide alpha-N-acetyltransferase |
| c290332_g1 | 216.66 | 101.11 | 1.10 | 0.00 | 3.43E-110 | E3.2.1.21 | beta-glucosidase |
| c339164_g1 | 111.90 | 55.08 | 1.02 | 0.02 | 0 | GPI, pgi | glucose-6-phosphate isomerase |
| c174862_g1 | 66.15 | 140.79 | -1.09 | 0.04 | 1.93E-102 | DEAD | DEAD/DEAH box helicase domain-containing protein |
| c138588_g1 | 100.52 | 50.05 | 1.01 | 0.02 | 7.14E-53 | LHCA1 | light-harvesting complex I chlorophyll a/b binding protein 1 |
| c194583_g2 | 1525.24 | 710.90 | 1.10 | 0.00 | 0 | metH, MTR | 5-methyltetrahydrofolate--homocysteine methyltransferase |
| c177246_g1 | 71.69 | 30.59 | 1.23 | 0.02 | 9.80E-108 | msrA | peptide-methionine (S)-S-oxide reductase |
| c165998_g3 | 337.95 | 163.32 | 1.05 | 0.01 | 1.70E-173 | MYO5 | myosin V |
| c130034_g2 | 82.77 | 37.63 | 1.14 | 0.02 | 4.39E-55 | NAPA, SNAPA, SEC17 | alpha-soluble NSF attachment protein |
| c148854_g2 | 689.14 | 310.16 | 1.15 | 0.00 | 0 | NNT | NAD(P) transhydrogenase |
| c245961_g1 | 82.23 | 201.04 | -1.29 | 0.01 | 4.99E-61 | petC | cytochrome b6-f complex iron-sulfur subunit |
| c320355_g1 | 47.21 | 15.99 | 1.56 | 0.02 | 2.10E-54 | plsC | 1-acyl-sn-glycerol-3-phosphate acyltransferase |
| c312664_g1 | 187.44 | 77.75 | 1.27 | 0.00 | 1.01E-38 | PSMD8, RPN12 | 26S proteasome regulatory subunit N12 |
| c344356_g1 | 113.35 | 47.76 | 1.25 | 0.02 | 6.49E-11 | RP-L28, MRPL28, rpmB | large subunit ribosomal protein L28 |
| c337731_g1 | 27.20 | 91.56 | -1.75 | 0.02 | 2.06E-10 | RP-S16, MRPS16, rpsP | small subunit ribosomal protein S16 |
| c201849_g1 | 26.16 | 6.60 | 1.99 | 0.05 | 8.74E-51 | SACS | sacsin |
| c168946_g1 | 20.24 | 5.83 | 1.80 | 0.01 | 2.39E-84 | SLC25A14_30 | solute carrier family 25 (mitochondrial carrier), member 14/30 |
| c105137_g1 | 32.86 | 116.57 | -1.83 | 0.01 | 6.33E-16 | STIP1 | stress-induced-phosphoprotein 1 |
| c43525_g1 | 97.21 | 226.93 | -1.22 | 0.04 | 2.99E-32 | STIP1 | stress-induced-phosphoprotein 1 |
| c137145_g1 | 207.63 | 88.11 | 1.24 | 0.00 | 7.27E-152 | TM9SF3 | transmembrane 9 superfamily member 3 |
| c158471_g1 | 11.56 | 48.80 | -2.08 | 0.02 | 1.23E-37 | TRDMT1, DNMT2 | tRNA (cytosine38-C5)-methyltransferase |
| c202044_g1 | 1133.92 | 80.67 | 3.81 | 0.02 | 4.72E-15 | UBE2D_E, UBC4, UBC5 | ubiquitin-conjugating enzyme E2 D/E |
| c179578_g1 | 229.81 | 106.78 | 1.11 | 0.01 | 1.40E-82 | USP4_11_15, UBP12 | ubiquitin carboxyl-terminal hydrolase 4/11/15 |

**Table S7.** Comparison of the number of genes with transcription factor-related domains of *G. fascicularis* and other coral species. The number of proteins with Pfam domains are listed.

| **domain name** | **accession** | **description** | ***Galaxea fascicularis*** | ***Porites australiensis*** | ***Acropora digitifera*** | ***Nematostella vectensis*** | ***Hydra magnipapillata*** |
| --- | --- | --- | --- | --- | --- | --- | --- |
| HLH | PF00010 | Helix-loop-helix DNA-binding domain | 50 | 48 | 52 | 72 | 32 |
| Homeobox | PF00046 | Homeobox domain | 87 | 89 | 97 | 155 | 43 |
| Hormone_rece | PF00104 | Ligand-binding domain of nuclear hormone receptor | 16 | 12 | 9 | 21 | 7 |
| Pou | PF00157 | Pou domain - N-terminal to homeobox domain | 6 | 4 | 4 | 6 | 3 |
| bZIP_1 | PF00170 | bZIP transcription factor | 54 | 19 | 24 | 38 | 24 |
| Ets | PF00178 | Ets-domain | 14 | 15 | 12 | 16 | 9 |
| Fork_head | PF00250 | Fork head domain | 25 | 21 | 22 | 34 | 15 |
| PAX | PF00292 | 'Paired box' domain | 9 | 8 | 8 | 9 | 27 |
| SRF-TF | PF00319 | SRF-type transcription factor (DNA-binding and dimerisation domain) | 9 | 2 | 1 | 4 | 2 |
| GATA | PF00320 | GATA zinc finger | 13 | 2 | 5 | 5 | 5 |
| HMG_box | PF00505 | HMG (high mobility group) box | 0 | 24 | 26 | 35 | 33 |
| RHD | PF00554 | Rel homology domain (RHD) | 2 | 2 | 2 | 3 | 1 |
| DM | PF00751 | DM DNA binding domain | 11 | 3 | 8 | 12 | 6 |
| Runt | PF00853 | Runt domain | 1 | 0 | 1 | 1 | 1 |
| P53 | PF00870 | P53 DNA-binding domain | 3 | 3 | 3 | 3 | 2 |
| T-box | PF00907 | T-box | 6 | 8 | 10 | 16 | 7 |
| ARID | PF01388 | ARID/BRIGHT DNA binding domain | 8 | 7 | 5 | 6 | 7 |
| Basic | PF01586 | Myogenic Basic domain | 2 | 0 | 0 | 0 | 0 |
| AT_hook | PF02178 | AT hook motif | 5 | 1 | 0 | 0 | 0 |
| CUT | PF02376 | CUT domain | 3 | 3 | 1 | 2 | 0 |
| TF_AP-2 | PF03299 | Transcription factor AP-2 | 0 | 2 | 2 | 1 | 1 |
| TF_Otx | PF03529 | Otx1 transcription factor | 4 | 0 | 0 | 0 | 0 |
| GCM | PF03615 | GCM motif protein | 4 | 1 | 1 | 2 | 0 |
| OAR | PF03826 | OAR domain | 0 | 7 | 5 | 4 | 0 |
| Prox1 | PF05044 | Homeobox prospero-like protein (PROX1) | 3 | 0 | 0 | 0 | 0 |
| SIM_C | PF06621 | Single-minded protein C-terminus | 0 | 0 | 0 | 0 | 0 |
| Hairy_orange | PF07527 | Hairy Orange | 10 | 6 | 7 | 6 | 0 |
| P53_tetramer | PF07710 | P53 tetramerisation motif | 3 | 1 | 1 | 1 | 0 |
| bZIP_2 | PF07716 | Basic region leucine zipper | 37 | 15 | 17 | 34 | 20 |
| zf-C2H2 | PF00096 | Zinc finger, C2H2 type | 118 | 167 | 88 | 209 | 106 |
| zf-C4 | PF00105 | Zinc finger, C4 type (two domains) | 15 | 11 | 12 | 19 | 8 |
| zf-C2HC | PF01530 | Zinc finger, C2HC type | 6 | 4 | 4 | 4 | 2 |
| SCAN | PF02023 | SCAN domai | 6 | 1 | 4 | 0 | 0 |
| Total | | | 530 | 486 | 431 | 718 | 361 |

**Table S8. Comparison of the number of genes with cell signalling transduction-related domains of *G. fascicularis* and other coral species. The number of proteins with Pfam domains are listed.**

| **domain name** | **accession** | **description** | ***Galaxea fascicularis*** | ***Porites australiensis*** | ***Acropora digitifera*** | ***Nematostella vectensis*** | ***Hydra magnipapillata*** |
| --- | --- | --- | --- | --- | --- | --- | --- |
| MCPsignal | PF00015 | Methyl-accepting chemotaxis protein (MCP) signaling domain | 7 | 2 | 1 | 15 | 7 |
| wnt | PF00110 | wnt family | 11 | 15 | 15 | 29 | 11 |
| G-alpha | PF00503 | G-protein alpha subunit | 46 | 13 | 18 | 34 | 19 |
| RGS | PF00615 | Regulator of G protein signaling domain | 0 | 10 | 11 | 13 | 13 |
| G-gamma | PF00631 | GGL domain | 4 | 3 | 3 | 4 | 2 |
| HAMP | PF00672 | HAMP domain | 2 | 0 | 0 | 6 | 0 |
| DIX | PF00778 | DIX domain | 0 | 3 | 2 | 4 | 1 |
| STAT_alpha | PF01017 | STAT protein, all-alpha domain | 7 | 1 | 1 | 2 | 0 |
| CheW | PF01584 | CheW-like domain | 1 | 0 | 0 | 1 | 0 |
| Hpt | PF01627 | Hpt domain | 0 | 0 | 0 | 1 | 0 |
| Cbl_N | PF02262 | CBL proto-oncogene N-terminal domain 1 | 3 | 1 | 1 | 1 | 1 |
| Dishevelled | PF02377 | Dishevelled specific domain | 0 | 1 | 0 | 0 | 0 |
| Cbl_N2 | PF02761 | CBL proto-oncogene N-terminus, EF hand-like domain | 1 | 1 | 1 | 0 | 1 |
| Cbl_N3 | PF02762 | CBL proto-oncogene N-terminus, SH2-like domain | 0 | 1 | 1 | 0 | 1 |
| STAT_bind | PF02864 | STAT protein, DNA binding domain | 3 | 1 | 1 | 1 | 1 |
| STAT_int | PF02865 | STAT protein, protein interaction domain | 3 | 1 | 1 | 0 | 1 |
| NPH3 | PF03000 | NPH3 family | 0 | 0 | 0 | 0 | 0 |
| Focal_AT | PF03623 | Focal adhesion targeting region | 1 | 1 | 0 | 1 | 2 |
| Olfactory_mark | PF06554 | Olfactory marker protein | 1 | 0 | 0 | 0 | 0 |
| Phe_ZIP | PF08916 | Phenylalanine zipper | 3 | 1 | 1 | 0 | 0 |
| TRADD_N | PF09034 | TRADD, N-terminal domain | 2 | 0 | 0 | 0 | 0 |
| TGF_beta | PF00019 | Transforming growth factor beta like domain | 5 | 7 | 10 | 9 | 11 |
| FGF | PF00167 | Fibroblast growth factor | 9 | 14 | 13 | 14 | 13 |
| PDGF | PF00341 | Platelet-derived growth factor (PDGF) | 0 | 2 | 0 | 0 | 1 |
| TGFb_propepti | PF00688 | TGF-beta propeptide | 6 | 6 | 11 | 6 | 10 |
| IL2 | PF00715 | Interleukin 2 | 2 | 0 | 0 | 0 | 0 |
| IL4 | PF00727 | Interleukin 4 | 2 | 0 | 0 | 0 | 0 |
| PTN_MK_C | PF01091 | PTN/MK heparin-binding protein family, C-terminal domain | 3 | 0 | 0 | 0 | 0 |
| GM_CSF | PF01109 | Granulocyte-macrophage colony-stimulating factor | 1 | 0 | 0 | 0 | 0 |
| IL7 | PF01415 | Interleukin 7/9 family | 0 | 0 | 0 | 0 | 0 |
| IL5 | PF02025 | Interleukin 5 | 2 | 0 | 0 | 0 | 0 |
| IL3 | PF02059 | Interleukin-3 | 1 | 0 | 0 | 0 | 0 |
| IL12 | PF03039 | Interleukin-12 alpha subunit | 2 | 0 | 0 | 0 | 0 |
| Rabaptin | PF03528 | Rabaptin | 2 | 1 | 1 | 1 | 0 |
| PDGF_N | PF04692 | Platelet-derived growth factor, N terminal region | 0 | 0 | 0 | 0 | 0 |
| AMH_N | PF04709 | Anti-Mullerian hormone, N terminal region | 0 | 0 | 0 | 0 | 0 |
| PTN_MK_N | PF05196 | PTN/MK heparin-binding protein family, N-terminal domain | 2 | 0 | 0 | 1 | 0 |
| CSF-1 | PF05337 | Macrophage colony stimulating factor-1 | 1 | 0 | 0 | 0 | 0 |
| PSK | PF06404 | Phytosulfokine precursor protein (PSK) | 1 | 0 | 0 | 0 | 0 |
| IL11 | PF07400 | Interleukin 11 | 0 | 0 | 0 | 0 | 0 |
| Total | | | 134 | 85 | 92 | 143 | 95 |

**Table S9.** Abundance of vertebrate biomineralization-related and chitin-related domains among organisms. The number of proteins with Pfam domains are listed.

| **domain name** | **accession** | **description** | ***Galaxea fascicularis*** | ***Porites australiensis*** | ***Acropora digitifera*** | ***Nematostella vectensis*** | ***Hydra magnipapillata*** | ***Drosophila melanogaster*** | ***Strongylocentrotus purpuratus*** | ***Homo*** | ***Monosiga brevicollis*** | ***Emiliania huxleyi*** |
| --- | --- | --- | --- | --- | --- | --- | --- | --- | --- | --- | --- | --- |
| SPARC_Ca_bdg | PF10591 | Secreted protein acidic and rich in cysteine Ca binding region | 57 | 36 | 32 | 54 | 35 | 21 | 4 | 50 | 61 | 51 |
| DMP1 | PF07263 | Dentin matrix protein 1 (DMP1) | 2 | 0 | 0 | 0 | 0 | 1 | 0 | 1 | 0 | 0 |
| Osteopontin | PF00865 | Osteopontin | 1 | 0 | 0 | 0 | 1 | 0 | 0 | 1 | 0 | 0 |
| Gla | PF00594 | Vitamin K-dependent carboxylation | 1 | 1 | 0 | 0 | 0 | 0 | 0 | 14 | 0 | 0 |
| BSP_II | PF05432 | Bone sialoprotein II (BSP-II) | 0 | 0 | 0 | 1 | 0 | 0 | 0 | 3 | 0 | 0 |
| Osteoregulin | PF07175 | Osteoregulin | 0 | 0 | 0 | 0 | 0 | 0 | 0 | 1 | 0 | 0 |
| Amelogenin | PF02948 | Amelogenin | 7 | 0 | 0 | 0 | 0 | 0 | 0 | 2 | 0 | 0 |
| Amelin | PF05111 | Ameloblastin precursor | 0 | 0 | 0 | 0 | 0 | 0 | 0 | 1 | 0 | 0 |
| UnbV_ASPIC | PF07593 | ASPIC and UnbV | 0 | 2 | 0 | 0 | 0 | 0 | 3 | 1 | 0 | 9 |
| Carb_anhydrase | PF00194 | Eukaryotic-type carbonic anhydrase | 0 | 9 | 11 | 8 | 26 | 15 | 2 | 20 | 0 | 4 |
| Total | | | 68 | 48 | 43 | 63 | 62 | 37 | 9 | 94 | 61 | 64 |

**Table S10.** Abundance of innate immunity-related and apoptosis-related domains among organisms. The number of proteins with Pfam domains are listed.

| **domain name** | **accession** | ***Galaxea fascicularis*** | ***Porites australiensis*** | ***Acropora digitifera*** | ***Nematostella vectensis*** | ***Homo*** |
| --- | --- | --- | --- | --- | --- | --- |
| BIR | PF00653 | 0 | 3 | 4 | 3 | 8 |
| Bcl-2 | PF00452 | 12 | 8 | 8 | 13 | 11 |
| TNF | PF00229 | 11 | 20 | 16 | 6 | 22 |
| TNFR_c6 | PF00020 | 37 | 19 | 25 | 14 | 22 |
| DED | PF01335 | 21 | 33 | 31 | 13 | 7 |
| Peptidase_C14 | PF00656 | 22 | 16 | 32 | 14 | 14 |
| MATH | PF00917 | 10 | 41 | 36 | 25 | 12 |
| CARD | PF00319 | 12 | 16 | 38 | 11 | 28 |
| Death | PF00531 | 68 | 82 | 147 | 20 | 33 |
| NACHT/NB-ARC | (PF05729/PF00931) | 96 | 141 | 496 | 56 | 49 |
| Total | | 289 | 379 | 833 | 175 | 206 |

**Table S11. Abundance of candidate genes associated with symbiosis from *G. fascicularis* and other cnidarian sequences. The number of genes associated with symbiosis are listed.**

| **Gene name** | **Corals** | | | | | | | | **Anemones** | | |
| --- | --- | --- | --- | --- | --- | --- | --- | --- | --- | --- | --- |
|  | ***G. fascicularis*** | ***A. digitifera*** | ***A. hyacinthus*** | ***A. millepora*** | ***A. palmata*** | ***M. faveolata*** | ***P. astreoides*** | ***P. damicornis*** | ***N. vectensis*** | ***A. pallida*** | ***A. viridis*** |
| **PATTERN RECOGNITION** | | | | | | | | | | | |
| Lectins: C-type | 0 | 9 | 0 | 1 | 3 | 1 | 3 | 3 | 9 | 7 | 3 |
| Mannose receptor MR1 | 7 | 8 | 2 | 4 | 3 | 0 | 4 | 4 | 4 | 7 | 2 |
| Mannan-binding serine peptidase 2 (MASP2) | 1 | 2 | 2 | 1 | 3 | 1 | 2 | 1 | 0 | 1 | 0 |
| Complement component C3 | 1 | 3 | 2 | 5 | 1 | 1 | 5 | 2 | 2 | 2 | 3 |
| Toll/TLR | 5 | 8 | 5 | 8 | 4 | 0 | 3 | 6 | 6 | 3 | 2 |
| TNFR-associated (Traf6) | 6 | 11 | 4 | 5 | 13 | 2 | 6 | 11 | 8 | 10 | 7 |
| Scavenger receptor class B | 2 | 3 | 3 | 6 | 5 | 0 | 4 | 2 | 2 | 1 | 0 |
| Thrombospondin 1 | 3 | 11 | 11 | 12 | 9 | 1 | 11 | 11 | 20 | 11 | 4 |
| NOD-like receptors: NACHT | 12 | 10 | 9 | 6 | 9 | 1 | 7 | 18 | 5 | 3 | 10 |
| **CELL ADHESION** | | | | | | | | | | | |
| Fasciclin I (Sym32/periostin) | 1 | 2 | 1 | 2 | 2 | 1 | 2 | 1 | 3 | 1 | 1 |
| γ-Glutamylcarboxylase | 3 | 1 | 2 | 2 | 1 | 2 | 1 | 1 | 2 | 3 | 1 |
| Vitamin K epoxide reductase | 1 | 1 | 1 | 1 | 1 | 1 | 1 | 0 | 2 | 0 | 1 |
| Calumenin | 9 | 2 | 4 | 4 | 14 | 5 | 4 | 4 | 7 | 7 | 6 |
| **VESICULAR TRAFFICKING** | | | | | | | | | | | |
| Rab GTPases | 41 | 26 | 36 | 38 | 55 | 15 | 32 | 16 | 41 | 22 | 17 |
| NADPH oxidase | 5 | 3 | 4 | 4 | 5 | 0 | 2 | 3 | 5 | 10 | 1 |
| Vacuolar H+ATPase | 16 | 9 | 16 | 15 | 32 | 11 | 16 | 31 | 15 | 15 | 4 |
| Autophagy-specific (Atg8) | 1 | 4 | 4 | 3 | 5 | 3 | 3 | 3 | 4 | 4 | 5 |
| **REGULATION OF INCOMING LIGHT** | | | | | | | | | | | |
| Dehydroquinate synthase | 0 | 1 | 0 | 0 | 3 | 0 | 1 | 2 | 2 | 1 | 1 |
| *O*-Methyltransferase | 8 | 1 | 3 | 2 | 3 | 1 | 1 | 2 | 2 | 2 | 1 |
| ATP-grasp | 0 | 1 | 0 | 0 | 0 | 0 | 0 | 0 | 1 | 1 | 0 |
| Nonribosomal peptide synthetase | 2 | 1 | 2 | 3 | 5 | 0 | 1 | 0 | 3 | 3 | 2 |
| Fluorescent proteins | 11 | 8 | 2 | 3 | 5 | 1 | 3 | 2 | 2 | 0 | 3 |
| **APOPTOSIS** | | | | | | | | | | | |
| BCL2-associated X (Bax) | 3 | 7 | 3 | 5 | 8 | 6 | 4 | 6 | 7 | 5 | 4 |
| Caspase 8 | 4 | 9 | 3 | 5 | 11 | 1 | 5 | 11 | 6 | 12 | 4 |
| Sphingosine kinase 1 | 1 | 2 | 2 | 1 | 4 | 2 | 2 | 2 | 4 | 6 | 0 |
| Sphingosine-1-phosphate Phosphatase 1 | 2 | 1 | 2 | 5 | 2 | 0 | 3 | 1 | 4 | 3 | 3 |
| Nitric oxide synthase | 3 | 2 | 0 | 2 | 2 | 0 | 1 | 1 | 1 | 2 | 0 |
| TGF β | 4 | 7 | 0 | 1 | 6 | 0 | 2 | 5 | 6 | 6 | 3 |
| **NUTRIENT AND METABOLITE TRANSPORT** | | | | | | | | | | | |
| P-type H + ATPase | 8 | 8 | 14 | 13 | 19 | 1 | 14 | 11 | 16 | 20 | 6 |
| Carbonic anhydrase | 18 | 8 | 7 | 8 | 14 | 1 | 7 | 11 | 7 | 6 | 5 |
| Aquaporin | 8 | 2 | 2 | 3 | 4 | 1 | 1 | 8 | 8 | 9 | 0 |
| ABC transporters | 52 | 34 | 33 | 35 | 30 | 6 | 39 | 32 | 69 | 46 | 33 |
| Glutamate dehydrogenase | 2 | 3 | 6 | 3 | 12 | 1 | 5 | 12 | 4 | 4 | 9 |
| Glutamine synthetase | 8 | 1 | 2 | 5 | 4 | 1 | 5 | 3 | 2 | 2 | 2 |
| **LIPID STORAGE AND TRANSPORT** | | | | | | | | | | | |
| Perilipin (PAT) | 0 | 0 | 0 | 0 | 0 | 0 | 0 | 0 | 1 | 1 | 1 |
| NPC1 | 3 | 1 | 2 | 2 | 6 | 4 | 3 | 10 | 3 | 9 | 4 |
| NPC2 | 0 | 5 | 5 | 3 | 7 | 5 | 2 | 3 | 3 | 6 | 7 |
| **REPONSE TO ROS** | | | | | | | | | | | |
| Superoxide dismutase | 2 | 2 | 2 | 2 | 5 | 2 | 1 | 4 | 4 | 3 | 1 |
| Catalase | 1 | 1 | 5 | 4 | 3 | 1 | 3 | 3 | 2 | 4 | 2 |
| Peroxiredoxin | 6 | 3 | 3 | 3 | 7 | 5 | 1 | 9 | 3 | 4 | 5 |
| Glutathione peroxidase | 6 | 4 | 3 | 5 | 7 | 4 | 5 | 3 | 9 | 6 | 6 |
| Glutathione reductase | 1 | 1 | 2 | 1 | 6 | 1 | 5 | 1 | 1 | 1 | 2 |
| γ-Glutamylcysteine synthetase | 1 | 1 | 3 | 3 | 1 | 1 | 1 | 1 | 1 | 1 | 1 |
| Glutathione synthase | 1 | 2 | 5 | 4 | 2 | 0 | 3 | 3 | 1 | 2 | 0 |
| Glutathione S-transferase | 10 | 1 | 4 | 5 | 7 | 5 | 3 | 8 | 8 | 3 | 6 |
| Ferritin | 2 | 2 | 1 | 1 | 5 | 5 | 1 | 8 | 5 | 2 | 3 |
